# Ecomorphology of Neotropical Electric Fishes: An Integrative Approach to Testing the Relationships between Form, Function, and Trophic Ecology

**DOI:** 10.1093/iob/obz015

**Published:** 2019-07-02

**Authors:** K M Evans, L Y Kim, B A Schubert, J S Albert

**Affiliations:** 1 College of Food, Agricultural and Natural Resource Sciences, University of Minnesota, 1987 Upper Buford Circle, St. Paul, MN 55108, USA; 2 Department of Biology, University of Louisiana at Lafayette, P.O. Box 42451, Lafayette, LA 70504, USA; 3 School of Geosciences, University of Louisiana at Lafayette, P.O. Box 43705, Lafayette, LA 70504, USA

## Abstract

The relationship between form and function is thought to play an integral role in structuring broad-scale patterns of morphological evolution and resource utilization. In ecomorphological studies, mechanical performance is widely understood to constrain the evolution of form and function. However, the relationship between form, function, and resource utilization is less clear. Additionally, seasonal fluctuations in resource availability may further complicate patterns of resource use. How organisms cope with these complexities, and the effect of these factors on broadscale patterns of morphological evolution is also poorly understood. Here we use three-dimensional geometric morphometrics, biomechanics, stable isotope analysis, and gut-content analysis to study trophic evolution in a clade of riverine-adapted electric fishes from a region with high seasonal variability; the Amazon River. We find significant and phylogenetically structured relationships among measures of trophic ecology and skull shape. We also recover a significant relationship between the mechanical advantage of the mandible and trophic position, where species feeding at higher trophic levels have narrower jaws with lower mechanical advantages, and species feeding at lower trophic levels have deeper jaws with higher mechanical advantages. Our results indicate that selection is driving the evolution of mandible shape and performance toward specialization on different trophic ecologies.

## Synopsis

Ecomorfologia de Peixes Elétricos Neotropicais: Uma Abordagem Integrativa para Testar as Relações Entre Forma, Função, e Ecologia Trófica Acredita-se que a relação entre forma e função desempenhe um papel integral na estruturação de padrões de larga-escala de evolução morfológica e utilização de recursos. Em estudos ecomorfológicos, se é amplamente atribuído ao desempenho mecânico a restrição da evolução da forma e da função. Em estudos de sistemas complexos, como mandíbulas de peixes, a ideia de que a morfologia está relacionada ao desempenho é frequentemente estabelecida, mas sua relação com a utilização de recursos é menos clara. Aqui, nós usamos morfometria geométrica tridimensional, biomecânica, análise de isótopos estáveis e análise de conteúdo intestinal para estudar a evolução trófica em um clado de peixes elétricos adaptados a rios, da região da bacia Amazônica. Encontramos relações significativas e estruturadas filogeneticamente entre medidas de ecologia trófica e forma do crânio. Também recuperamos uma relação significativa entre a vantagem mecânica da mandíbula e posição trófica, onde as espécies que se alimentam em níveis tróficos superiores têm mandíbulas mais estreitas com menores vantagens mecânicas, e as espécies que se alimentam em níveis tróficos inferiores têm mandíbulas mais profundas com maiores vantagens mecânicas. Nossos resultados indicam que a seleção está conduzindo a evolução da forma e desempenho da mandíbula para a especialização em diferentes ecologias tróficas. Translated to Portuguese by J.P. Fontenelle (jp.fontenelle@mail.utoronto.ca)

Ökomorphologie Neotropischer Elektrofische: Ein Integrativer Ansatz zur Überprüfung der Beziehungen Zwischen Form, Funktion, und Trophischer Ökologie Es wird angenommen, dass die Beziehung zwischen Form und Funktion eine wesentliche Rolle bei der Strukturierung von morphologischen Evolutionsmustern sowie Ressourcennutzungsmustern spielt. In ökomorphologischen Studien wird allgemein davon ausgegangen, dass mechanische Leistung die Entwicklung von Form und Funktion einschränkt. In Studien über komplexe Systeme wie Fischkiefer wird häufig die Vorstellung etabliert, dass Morphologie mit der Leistung zusammenhängt, deren Beziehung zur Ressourcennutzung ist jedoch weniger klar. Hier verwenden wir dreidimensionale geometrische Morphometrie, Biomechanik, Analyse stabiler Isotope und Analyse des Darminhalts, um die trophische Evolution in einer Klade von Fluss-adaptierten Elektrofischen aus dem Amazonas-Becken zu untersuchen. Wir finden signifikante und phylogenetisch strukturierte Beziehungen zwischen Messgrößen der trophischen Ökologie und der Schädelform. Wir stellen auch eine signifikante Beziehung zwischen dem Unterkiefer als Kraftwandler und der Trophiebene her, wo Arten, die sich auf höheren trophischen Ebenen ernähren, schmalere Kiefer mit schlechterer Kraftwandlung haben und Arten, die auf niedrigeren trophischen Ebenen fressen, tiefere Kiefer mit besserer Kraftwandlung haben. Unsere Ergebnisse zeigen, dass die Selektion die Evolution von Form und Leistungsfähigkeit des Unterkiefers in Richtung einer Spezialisierung auf verschiedene trophische Ökologien treibt. Translated to German by F. Klimm (frederike.klimm@biologie.uni-freiburg.de)

## Introduction

Trophic ecology is thought to be a strong driver of morphological diversification in teleost fishes, with shifts to novel behaviors and prey items exerting differential selective pressures on feeding morphologies and performance (Wainwright et al. 1991, 2004; Westneat 2005; Collar et al. 2014). Within the skull of teleostean fishes, the neurocranium and mandible play important roles in prey capture and processing. As a result, these structures have become widely used models for ecomorphological studies linking the shape and ecological performance of musculo-skeletal structures (Motta 1988; Wainwright et al. 1991; Liem 1993; Westneat 2005). Studies estimating the mechanical performance of the mandible in particular have shown that variation in mechanical advantage is tightly linked to variation in trophic ecology and resource use. Species with higher mechanical advantages have been shown to feed on harder prey items while species with lower mechanical advantages have been shown to feed on more evasive prey (Wainwright et al. 1991, 2004; Westneat 2004; Konow et al. 2017).

While mechanical performance is expected to closely track resource utilization (Wainwright and Reilly 1994), the relationship between underlying morphologies and resource utilization is less predictable. This lack of predictability may be due in part to functional redundancy, or “many-to-one mapping” (Alfaro et al. 2005; Wainwright et al. 2005). Functional redundancy occurs when several different underlying morphologies are capable of producing similar performance outputs. The four-bar linkage system that powers jaw protrusion in teleost fishes is a widely-cited example of a functionally redundant system (Alfaro et al. 2005; Wainwright et al. 2005). In this system, differing linkage lengths can produce similar performance outputs, which themselves, often, more closely track measures of trophic ecology. This functional redundancy may therefore weaken the relationship between morphology and resource use.

Here we study the relationships among trophic morphology, mechanical performance, and trophic ecology in Navajini, a clade of riverine-adapted Neotropical electric fishes (Gymnotiformes: Teleostei). Gymnotiform fishes are a moderately diverse (∼300 spp.) clade of weakly electric fishes which exhibit elongate eel-like bodies and all possess elongate anal fins for use in a specialized form of locomotion (Albert 2001; van der Sleen and Albert 2017; Evans et al. 2018b). In addition to their derived body shapes, gymnotiform fishes also exhibit a diversity of skull shapes and trophic ecologies, ranging from fishes with elongate tube-snouts that probe the substrate and interstitial spaces to extract aquatic invertebrates, to brachycephalic piscivores who feed primarily on scales and tails of other electric fishes (Marrero and Winemiller 1993; Lundberg et al. 1996; Albert 2001; Albert and Crampton 2005; Bernt and Albert 2017; Evans et al. 2017a). Gymnotiformes have been the subject of several recent studies examining the developmental and evolutionary history of the skull and oral jaws (Evans et al. 2017b, 2017c, 2018a). However, to date, only a handful of studies have examined the relationships between morphology and trophic ecology in gymnotiform fishes (Marrero and Winemiller 1993; Winemiller and Adite 1997), and none have incorporated performance estimates or examined these relationships within a rigorous phylogenetic framework. The Navajini (Gymnotiformes: Apteronotidae) is a clade adapted to inhabit the deep channels (>5 m depth at mid-channel) of large, lowland Amazonian rivers (Albert 2003; Bernt and Albert 2017).

In addition to high levels of trophic diversity, Navajine fishes also inhabit a temporally variable aquatic environment in the Amazonian river channel (Crampton 2011), which can dramatically affect their foraging strategies. During the course of the annual hydrological cycle, the river and surrounding floodplains experience dramatic seasonal variation in water levels (Winemiller 1990; Albert and Reis 2011). During the period of high-water, rivers flood their banks and expand into the surrounding floodplain forest habitats, bringing fishes into contact with abundant allochthonous resources (e.g., terrestrial arthropods and fruits). During the low-water period, fishes lose contact with the floodplain and are restricted to habitats of the main river channels. As fishes become concentrated in river channels their densities increase (per unit surface area). As levels of dissolved oxygen decrease and water quality deteriorates, interactions between species intensify as species compete for limited resources. During this period, some Neotropical fishes have been shown to exhibit facultative lepidophagy (scale-feeding); presumably to exploit the increased fish densities in the restricted river channels, and switch to invertivory during high water periods (Peterson and Winemiller 1997; Peterson and McIntyre 1998). This seasonal variation in habitat quality and availability exerts strong selective pressures on species that inhabit large rivers and floodplains of lowland Amazonia (Val and de Almeida-Val 2012). Species that inhabit these habitats must either specialize on exploiting resources during a particular period of the seasonal variation (e.g., invertivory and lepidophagy), or adapt to exploit a variety of resources (e.g., generalist) as they change in relative availability.

In this study, we investigate the relationship between skull morphology, performance, and trophic ecology. We implement an integrative approach, combining three-dimensional geometric morphometrics, biomechanics, stable isotope analysis, gut-content analysis, and phylogenetic comparative methods to model the ecomorphological interactions of the morphology, mechanical performance, and trophic ecology of 11 species of Navajine electric fishes. We fit these data to alternative models of trait evolution to test the hypothesis of performance evolution via stabilizing selection at a single trait optimum, multiple trait optima, or a neutral drift model.

We hypothesize that skull shape and mandible performance closely predict trophic ecology. We further hypothesize that species evolve to specialize on specific trophic resources, resulting in the evolution of distinct performance strategies in the Amazon River.

## Materials and methods

### CT-scanning and skull shape geometric morphometrics

**Fig. 1:**
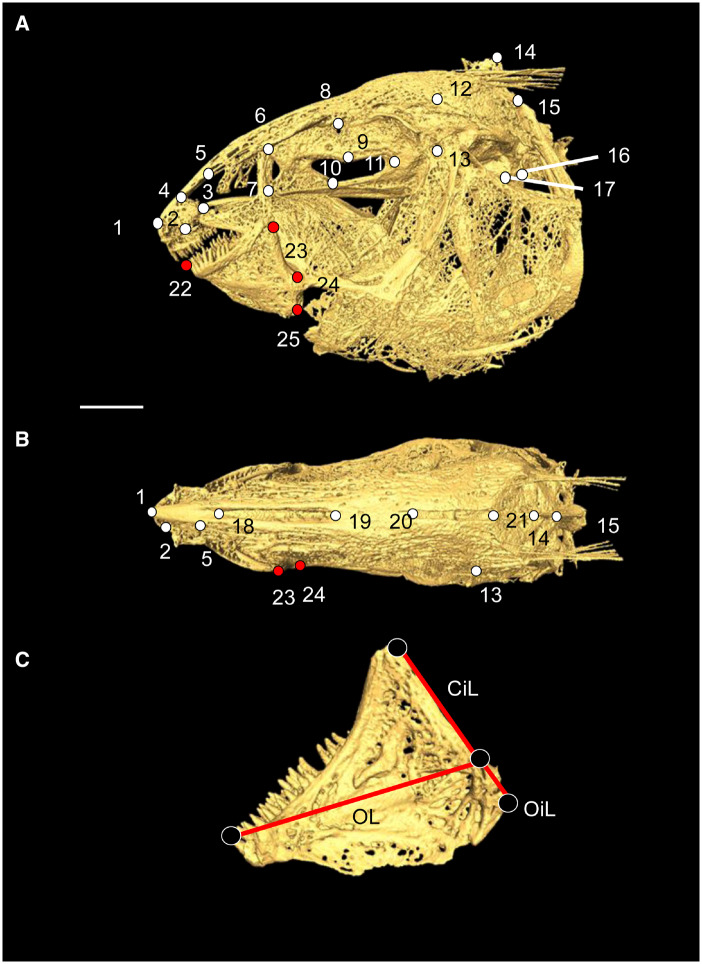
Three-dimensional skull surface rendering of *Sternarchella orinoco* showing the location of 25 landmarks colored to correspond to neurocranium (white), and mandible (red) regions, in lateral (**A**) and dorsal (**B**) views. **C**) Linear distances used to calculate mechanical advantage measurements on the mandible. Scale bar=5.0 mm.

Craniofacial shape was characterized across an assemblage of 11 Navajine species (44% taxon sampling; Supplementary Table S1) from the Western region of the Amazon River in Iquitos, Peru. An average of five adult specimens per species were micro-CT scanned at the University of Washington Friday Harbor Labs (UW) Karl Liem Memorial Bio-Imaging Facility in conjunction with the “ScanAllFishes” project using a Bruker Skyscan 1173 at 70 kV, 114 μA, and 20.2–35.0 μm voxel size. Sampling for all analyses was restricted to adult specimens (as evidenced by body size and degree of sphenoid ossification) to avoid potential biases introduced by ontogenetic shape differences (Evans et al. 2017b). Micro-CT scans were then used to construct surface models (.stl) for each specimen using *Amira* and imported into *Stratovan Checkpoint* for digitizing. All three-dimensional models are freely available for download at osf.io/q4aw5. Specimens were digitized in three dimensions with 25 landmarks placed on the left side of each specimen (Fig. 1A, B andSupplementary Table S2) following the approach of Evans et al. (2018a). The raw landmark data were then imported into *MorphoJ* (Klingenberg 2011) where a full Procrustes superimposition was performed on the data to correct for differential scaling and orientation of specimens. A principal components (PCs) analysis was conducted to quantify principal axes of shape variation among the data. The PC scores were then used to construct a phylomorphospace using the first two PCs which explained 68.1% of the total shape variance (Sidlauskas 2008), using the phylogeny of Tagliacollo et al. (2016). Procrustes coordinates were then imported into the r-package *geomorph* (Adams and Otarola-Castillo 2013; R core team 2018) for all subsequent analyses.

### Phylogenetic tree

**Fig. 2:**
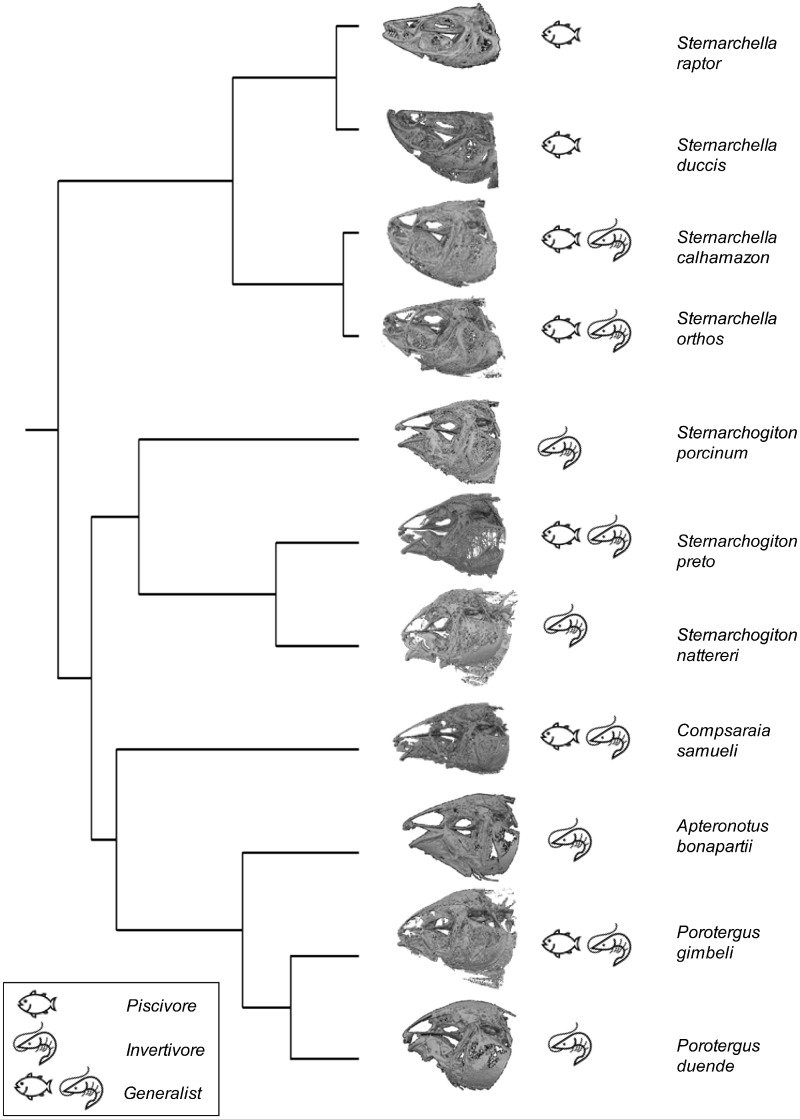
Phylogeny of the 11 species of Navajini used in this analysis from Tagliacollo et al. (2016). CT scans depict representative skull shapes for each species. Fish and shrimp insets depict trophic ecology (as estimated in this study) for each species.

To study the evolution of craniofacial shape across deep-channel electric fishes, we used a recently published phylogeny for 133 gymnotiform species (Tagliacollo et al. 2016; Evans et al. 2017c). The phylogeny was pruned in the r-package *ape* (Paradis et al. 2004) to include the 11 species examined in this study (Fig. 2).

### Phylogenetic signal

To quantify the phylogenetic structure of our shape data, we used the multivariate K-statistic, which is optimized to analyze datasets with high-dimensionality (Adams 2014a). For trophic position (TP), we used Blomberg’s *K* (Blomberg et al. 2003) using the *phylo.sig* function implemented in *phytools* (Revell 2012).

### Estimating performance of the mandible

Performance was estimated by two measures of kinematic transmission in the mandible: closing and opening mechanical advantage (OMA) (Wainwright et al. 1991; Westneat 1994; Westneat 2004; Fig. 2C). Mechanical advantage is a widely-used ecomorphological predictor of displacement and force transmission in the lower jaw of teleost fishes (Westneat 2003; Kolmann et al. 2018). Additionally, differences in mechanical advantage are readily interpretable due to a well-documented trade-off where high values correspond to higher force outputs and lower relative velocity transmissions (assuming a fixed rate of actuator shortening; *sensu* [McHenry and Summers 2011]) while lower values correspond to lower force outputs and higher relative velocity transmissions. Mechanical advantage of the mandible can be modeled as simple third-order lever system with a single input and output lever, where mechanical advantage is measured as the ratio between the length of the input lever and the length of the output lever (Westneat 1994; Kolmann et al. 2018). Closing mechanical advantage (CMA) was measured as CMA = (CiL/OL) where CiL is the closing in-lever distance between the jaw joint and the dorsal insertion of the A2 adductor mandibulae muscular sub-unit and OL is the out lever distance between the jaw joint and the most anterior tooth on the dentary. OMA was measured as (OMA = OiL/OL) where OiL is the opening in-lever distance between the center of the jaw joint and the site of attachment of the interopercle ligament onto the posteroventral corner of the mandible or retroarticular (Fig. 1C). This model, while informative, has its limitations in that it underestimates force transmissions because it does not take into account muscle cross-sectional area (CSA) and attachment angle, and only considers the subdivision of the A2 moment arm in the case of CMA (Westneat 2003).

### Quantifying trophic ecology

To quantify TP among deep-channel electric fishes, we used stable isotope analysis. Muscle tissue samples about 1 cm^3^ were excised from the dorsal lateral surface of 78 specimens spanning 11 Navajini species collected by night trawls in the Iquitos, Peru area (03° 42.790′ S, 073° 13.952′ W) during the low water period (August 2015 and 2016). Specimens were dehydrated in NaCl at ambient temperature following Lujan et al. (2011). Skin and scales were removed prior to excision of muscle tissue to maintain homogeneity of sample. Voucher specimens were individually tagged and preserved in 10% unbuffered formalin for 24 h, washed twice in tap water to remove the formalin and then transferred to 70% ethanol for long term storage. All voucher specimens were cataloged at the Academy of Natural Sciences in Philadelphia.

Muscle tissue samples were dried at 60°C for 48 h, ground to a fine powder using a mortar and pestle, and weighed in tin capsules in preparation for stable isotope analyses. Samples were analyzed in triplicate for natural abundance stable isotope composition (δ^13^C or δ^15^N) using a Delta V Advantage Isotope Ratio Mass Spectrometer (Thermo Fisher, Bremen, Germany) configured to a Thermo Finnigan 1112 Series Flash Elemental Analyzer in the laboratory of BAS at the University of Louisiana at Lafayette. All data are reported in standard δ-notation (in units of permil, ‰) and expressed relative to international standards for carbon (Vienna Pee Dee Belemnite, VPDB) or nitrogen (mean air, AIR). Data were normalized to the VPDB and AIR scales using internal lab reference materials (JPEP: δ^13^C =−14.42‰, δ^15^N = +5.92‰; JALA: δ^13^C = −20.65‰, δ^15^N = −3.16‰; JGLY: δ^13^C = −43.51‰; USGS41: δ^13^C = +37.63‰, δ^15^N = +47.57‰). A quality assurance sample (JGLUT: δ^13^C =−13.43‰, δ^15^N =−4.34‰) was analyzed as an unknown with each batch run. Across all analyses, the JGLUT quality assurance sample averaged δ^13^C =−13.55 ± 0.09 (*n* = 11) and δ^15^N =−4.49 ± 0.15 (*n* = 11).

TP was estimated using the δ^15^N value, as it becomes more enriched as trophic level increases (Vander Zanden et al. 1997). TP was calculated as TP = (((δ^15^N fish −δ^15^N reference)/2.8)+1), where 2.8 refers to the estimated (%) mean trophic enrichment for tropical fishes (Jepsen and Winemiller 2002). The reference value in this equation is usually calculated as the mean value of periphyton or organic-rich sediment for a system. Periphyton was less frequent during the low water season, therefore, our reference was calculated using mean organic sediment δ^15^N values collected from river benthos. Primary production source was estimated using δ^13^C value. The mean ratio of C:N (by mass) was found to be higher than 3.5 indicating high lipid content (Burress et al. 2016). Lipid content is known to affect the δ^13^C value of bulk tissue due to its depletion in ^13^C relative to carbohydrates and proteins (Post et al. 2007). To correct for this potential bias, all δ^13^C values are reported as normalized values using the Post et al. (2007) equation for aquatic organisms, where δ^13^C normalized = δ^13^C untreated − 3.32 + 0.99 × C:N.

An analysis of variance (ANOVA) was used to test for significant effects of species on TP and carbon source. Tukey’s honest significant difference (HSD) pairwise comparison was performed *post hoc* to test for pairwise comparisons between species while taking into account differences in sample size and variance. All statistical analyses were performed in R version 3.2 (R Core Team) using the *Mass* version 7.3 package (Ripley and Venables 2013).

### Gut-content analysis

In conjunction with our stable isotope data, we used gut-content analysis to obtain a high-resolution (but temporally restricted) snapshot of specific food items ingested by the different species. Ninety-six specimens (representing 11 species) were collected concurrently with the specimens used in the stable isotope analysis using the same collection and preservation methods (see above). The entire GI tract was dissected out from each specimen using an Olympus SZX-12 stereomicroscope, and the contents of the foregut (esophagus and stomach) were removed and examined. Prey items were sorted and classified into three categories: aquatic invertebrates, fish-parts, and detritus. Unrecognizable contents were excluded from analyses. Volumetric proportions of prey items were estimated following the procedure of Winemiller (1990). Contents were spread on a slide and their area was compared to distilled water of a known volume.

### Effect of diet on skull shape

To quantify relationships between three-dimensional shape data, and TP, a phylogenetic Procrustes ANOVA (Collyer et al. 2015) was performed to account for the high dimensionality of the data. To evaluate the relationship at a finer scale, we subdivided the skull by analyzing subsets of landmarks in two regions: the neurocranium (landmarks 1–21) and the mandible (landmarks 22–25). We subdivided the skull to test for finer-scale relationships between different regions of the skull and diet, as both regions have been purported to play a direct or indirect role in prey capture and processing (Westneat 2004, 2005). Each landmark subset was subjected to a new Procrustes superimposition. We then used separate Procrustes ANOVAs to test for trophic relationships between the subdivisions of the skull. For this approach, only a BM error structure was employed (Adams 2014b). There is a growing literature about the challenges and hazards associated with fitting small (low *n*), highly dimensional multivariate datasets to OU models (Uyeda et al. 2015; Adams and Collyer 2017). As a result, we avoided fitting our raw shape data to OU models.

### Evaluating functional redundancy between performance and mandible shape

We also used a phylogenetic Procrustes ANOVA to test for the presence of functional redundancy between mechanical advantage and mandible shape (Procrustes shape coordinates). If functional redundancy was present in our data, we would expect to find an insignificant relationship between mandibular shape and performance estimates. A significant relationship would indicate that variation in mandibular shape has direct performance implications.

### Interface between performance and trophic ecology

The relationship between performance and ecology has the potential to yield valuable insights for the mapping of morphology to the external environment and resource utilization. However, due to the non-independent nature of species (such that species tend to resemble their closest relatives), it is important to consider the underlying phylogenetic structure of species included in any study (Felsenstein 1985). Relationships between estimated performance (closing and OMA) and TP were evaluated using a phylogenetic generalized least-squares (PGLS) regression (Rzhetsky and Nei 1992) using the pruned phylogeny of Evans et al. (2017c). The PGLS analysis allows for flexibility in the background error/covariance structure that can be fit to the data, as opposed to the standard Brownian motion (BM) error structure that is used for independent contrasts and the Procrustes PGLS (Martins and Hansen 1997; Adams 2014b). For this analysis, we fit our performance data to a BM error structure and an Ornstein–Uhlenbeck (OU) error structure. The OU model is an alternative to the simpler BM model in that it estimates the rate of adaptation (α) toward an adaptive optima (ϴ) (which is equal to 0 in the case of BM), while measuring random fluctuations in the evolutionary process (σ). Using the OU approach, investigators can assign multiple adaptive optima *apriori*, and estimate variable rates of selection toward these optima (Butler and King 2004; Uyeda and Harmon 2014; Maestri et al. 2017). For the PGLS analyses, performance traits were fit to a single-rate BM model (modeling neutral drift with a trait-specific rate of evolution) and a single-peak OU model (OU1) (stabilizing selection at a particular peak). Analyses were performed in the *r*-package *nlme* version 3.1 (Pinheiro et al. 2014).

We also evaluate performance evolution using more complex models in the *r*-package *mvMORPH* (Clavel et al. 2015). For this approach, we fit our performance data to several models consisting of different numbers of trait optima. Trait optima are defined following dietary categories that were binned using our continuous TP data into three discrete categories: invertivores (TP < 3.0) generalists (TP 3.0–3.8) and piscivores (TP 3.8–4.23). We estimated the ancestral state of TP using a Bayesian approach of stochastic character mapping following the approach of Bollback (2006) in the *R*-package: *phytools* (Revell 2012). Character history was modeled as a discrete trait under an “equal rates” transition matrix model using the Tagliacollo et al. (2016) phylogeny, pruned in the *r*-package *ape* (Paradis et al. 2004) to include only the 11 Navajini taxa. Transition frequencies of ancestral states were estimated from 1000 simulations creating 1000 different estimated character histories or SIMMAPs. Performance data were then fit to each of these SIMMAPs under a single peak OU model (OU1); a three-peak OU model (OU3) consisting of piscivores, generalists, and invertivores (Supplementary Fig. S1); a two-peak model (OU2) consisting of piscivores and invertivores (Supplementary Fig. S2); and a multi-rate Brownian motion model (BMM). Model support was assessed using a variation of the Akaike information criterion (AICc) which corrects for low sample sizes (Cavanaugh 1997). Strong model support (lowest AICc) for an OU1 model would indicate stabilizing selection on performance toward a single adaptive optima, model support for an multi-peak model would indicate the presence of multiple adaptive peaks in the trait data suggesting that different trophic ecologies are driving organismal performance toward specific adaptive peaks, and strong support for a BMM model would indicate no evolution toward adaptive optima (i.e., neutral drift).

To account for uncertainty in our model selection parameter estimations, we used a parametric bootstrapping approach that simulates data estimates under a known phylogeny and then re-estimates model fit for each simulated dataset (Boettiger et al. 2012). For both measures of mechanical advantage, we quantified the uncertainty in our model selection for the three-peak model (piscivores, generalists, and invertivores), the two-peak generalist-invertivore model, the two-peak piscivore-invertivore model, a single peak OU model, and the BM model by examining the percent of simulations in which the generating model was selected as the best fit. All parametric bootstrapping analyses were performed in the r-package *OUCH* (Butler and King 2004).

## Results

### Trophic ecology in Navajini

**Fig. 3:**
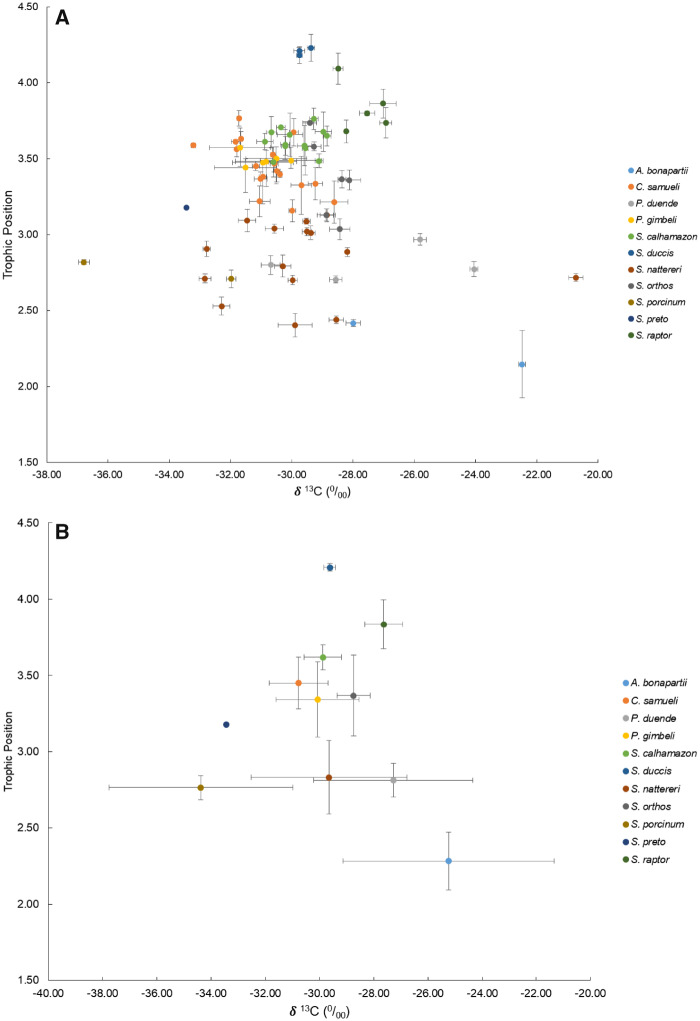
Trophic ecology of 11 species of Navajini. **A**) Mean (±standard deviation) trophic position (TP) plotted against δ^13^C for 78 specimens (232 samples) from the Amazon River near Iquitos, Peru. **B**) Mean (±standard deviation) TP plotted against δ^13^C for the 11 species.

The Navajini exhibit a wide diversity of trophic ecologies (Fig. 3). Species broadly overlap along the carbon-source (δ^13^C) axis. These overlapping values suggest that all Navajine species in this analysis belong to the same carbon source pool. This is an expected result, as all of the individuals in this analysis were collected from the same river channel habitat. Along the TP axis, species show an interesting stratification pattern. Pairwise differences in δ^13^C and TP are reported in Supplementary Table S3. A significant (*P* = 0.048) positive relationship was recovered between the proportion of fish remains in the gut and TP, such that species that incorporate more fish tissues into their diets feed at higher TPs. Species that feed primarily (>90% gut proportion) on aquatic arthropods (and detritus) (e.g., *Apteronotus bonapartii*, *P. duende*, and *Sternarchogiton* spp.) exhibit the lowest TPs, ranging from TP = 2.28 in *A. bonapartii* to 2.81 in *S. nattereri* (Table 1). We find that all species of *Sternarchella* incorporated some degree of fish tissue into their diets and likely as a result, exhibit higher TPs. We find that *S. duccis* and *S. raptor* feed exclusively on the scales and tails of other electric fishes. Generalist species (*excluding S. orthos*; incorporating both fish and aquatic invertebrate tissues into their diets) also exhibit some lepidophagy although to a lesser degree, as evidenced by lower proportions of fish tissues found in their guts.

**Table 1:** Diet proportions (±standard deviation) from gut-content analysis of 10 Navajini species from Iquitos, Peru

Species	Aquatic invertebrates	SD	Fish remains	SD	Detritus	SD	*n*
*A. bonapartii*	0.96	0.11	0.04	0.11	0.00	0.00	7
*C. samueli*	0.67	0.31	0.17	0.24	0.16	0.27	18
*P. duende*	1.00	0.00	0.00	0.00	0.00	0.00	9
*P. gimbeli*	0.75	0.22	0.06	0.14	0.21	0.18	6
*S. calhamazon*	0.84	0.24	0.11	0.22	0.05	0.09	27
*S. duccis*	0.01	0.01	0.39	0.27	0.60	0.28	4
*S. nattereri*	0.43	0.46	0.00	0.00	0.61	0.43	10
*S. orthos*	0.54	0.39	0.46	0.39	0.00	0.00	7
*S. porcinum*	0.70	0.27	0.00	0.00	0.30	0.27	5
*S. raptor*	0.00	0.00	1.00	0.00	0.00	0.00	3

*Note*: No contents were identified for *Sternarchogiton preto*, which was excluded from the analysis.

### Evolution of skull shape in channel fishes

**Fig. 4:**
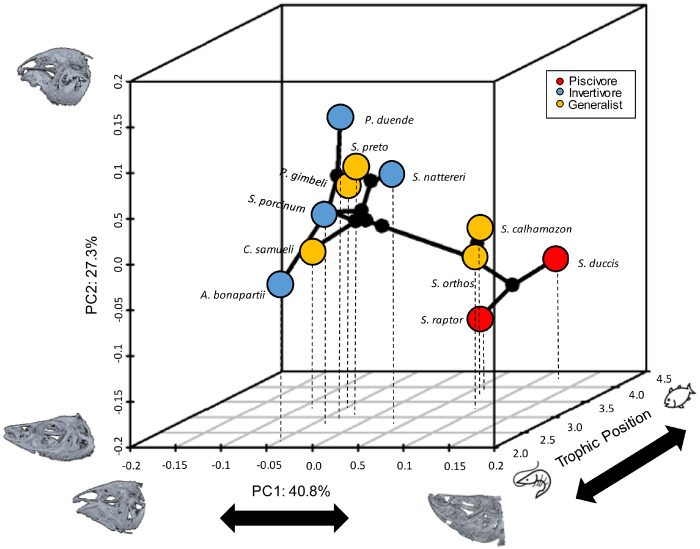
Evolution of ecology and morphology in Navajine fishes. Three-dimensional phylomorphospace analysis of 11 Navajini species showing primary axes of skull shape variation and TP with binned trophic ecologies plotted at each tip. Trophic ecologies were assigned to three different categories following mean TP for each species (Invertivore: 2.28–2.99, Generalist: 3.0–3.79, Piscivore: 3.8–4.21). Insets depict skull shape extremes for each axis.

Deep channel electric fishes display a wide diversity of craniofacial shapes, with much of the phenotypic disparity localized to the mandible region (Supplementary Fig. S3A, B and Fig. 4). The first PC axis of shape variation corresponds to differences in the shape of the posterior portion of the mandible encompassing the jaw joint and the retroarticular bone. In this region, shapes range from elongate and narrow (i.e., *Sternarchogiton porcinum*) to short and deep (i.e., *Sternarchella raptor*). Most upper jaw shape variation is confined to the second PC axis, where shapes range from more rounded skulls with a ventrally deflected facial region and a sub-terminal mouth position (e.g., *Porotergus duende*) to a more elongate and narrow skull with a dorsally inflected facial region and terminal to superior mouth position (e.g., *S. raptor*) (Fig. 4). Craniofacial shape exhibits significant phylogenetic signal within the Navajini (*P* = 0.003). Species of *Sternarchella* for example exhibit a more-slender mandible and cluster around the higher PC1 values of shape space, while the species *A.bonapartii* exhibits a deeper dentary and a more elongate posterior mandibular region (Fig. 4).

### Skull shape and TP

We find significant phylogenetic structure in both TP (*P* = 0.026) and skull shape (*P* < 0.001) within the Navajini. We also recover a significant (*P* = 0.011) relationship between skull shape and TP (Table 2), but is no longer significant with a phylogenetic Procrustes ANOVA (*P* = 0.344) indicating of the strong phylogenetic structure of these trait values (Table 3). This pattern is recurrent when testing for correlations in shape changes among subdivisions of the skull (neurocranium and mandible) and TP. Interestingly, prior to phylogenetic correction, we find that neurocranial shape more closely predicts TP than mandible shape (*r*^2^ = 0.41 vs. 0.28).

**Table 2:** Results of Procrustes ANOVA examining the effect of trophic position (TP) on skull shape and subsets of skull shape (neurocranium and mandible)

Module	SS	MS	*R*-sq	*F*	*Z*	*P*
Skull	0.05	0.05	0.27	3.41	2.16	**0.011**
Mandible	0.07	0.07	0.28	3.47	1.59	**0.032**
Neurocranium	0.04	0.04	0.41	6.38	2.90	**<0.001**

Bold values indicate statistical significance.

**Table 3:** Results of PGLS analysis examining the effect of TP on skull shape and subsets of skull shape (neurocranium and mandible)

Module	SS	MS	*R*-sq	*F*	*Z*	*P*
Skull	0.04	0.04	0.11	1.10	0.38	0.344
Mandible	0.12	0.12	0.20	2.23	1.14	0.122
Neurocranium	0.03	0.03	0.14	1.42	0.78	0.219

### Mandible shape and performance

Redundancy in the mapping of morphology to performance has the potential to weaken the relationship between these two variables. Using a Procrustes ANOVA, we find a significant (*P* = 0.009) relationship between mandible shape and CMA, but not OMA (*P* = 0.091). There is also no correlation between opening and CMA (*P* = 0.073) further suggesting a decoupling of the posterior region of the mandible from the anterior region and subsequent performances. Procrustes PGLS analysis shows neither CMA or OMA are significantly correlated with the evolution of mandible shape (*P* = 0.103, *P* = 0.516) this suggests the presence of strong phylogenetic structure in the case of CMA.

### The relationship between performance and diet

**Fig. 5:**
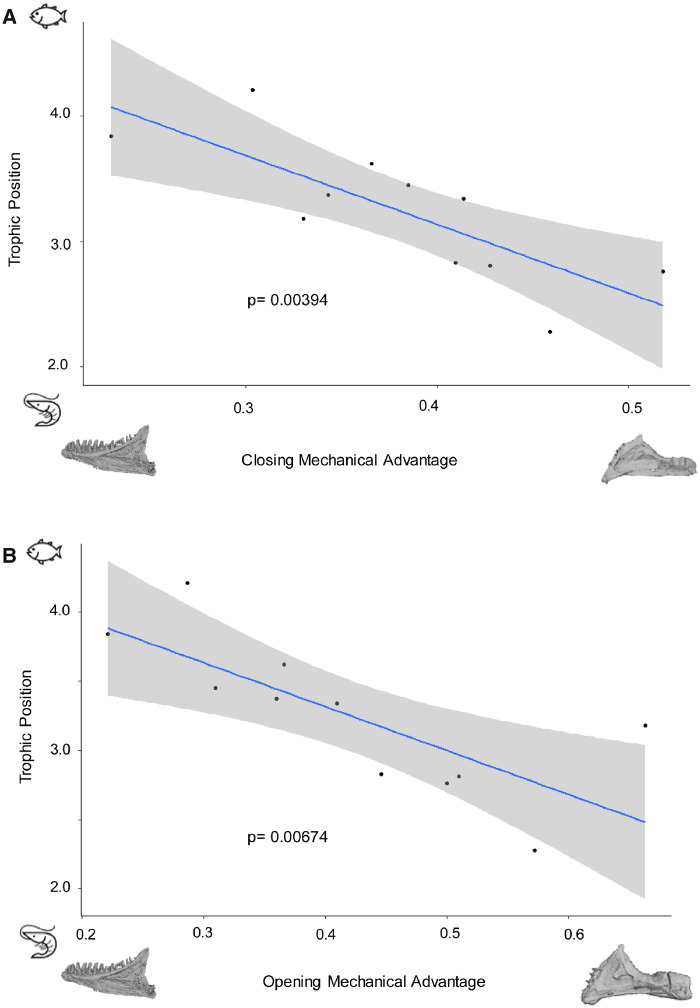
Relationship between functional performance of the mandible and trophic ecology. Closing (**A**) and opening (**B**) mechanical advantage vs. TP for 11 species of Navajini. Shaded regions denote 95% confidence intervals. Insets depict mandibular shape extremes for each functional performance measurement.

We find significant relationships between TP, and our two performance estimates: closing (*P* = 0.003) and opening (*P* = 0.006) mechanical advantage (Fig. 5). Among Navajini, species that feed at higher trophic levels typically exhibit lower mechanical advantages (i.e., mechanically fast jaws), while species that feed at lower TPs typically exhibit higher mechanical advantages (i.e., mechanically strong jaws). These relationships remain significant after PGLS analysis: closing (*P* = 0.006) and opening (*P* = 0.008) mechanical advantage (Table 4) when using an OU1 covariance structure.

**Table 4:** Results for PGLS analyses of CMA and OMA, showing AIC and BIC for OU and BMMs

Performance variable	Model	Slope	Intercept	AIC	BIC	Log-likelihood	*P*-value
Closing mechanical advantage	BM	0.44127	−0.015951	−13.75	−13.16	9.87	0.891
	OU	0.74711	−0.113003	−19.34	−18.75	12.67	**0.003**
Opening mechanical advantage	BM	0.58722	−0.051066	−5.64	−5.05	5.82	0.822
	OU	1.01206	−0.181796	−8.13	−7.53	7.06	**0.008**

Bold values indicate statistical significance.

**Fig. 6:**
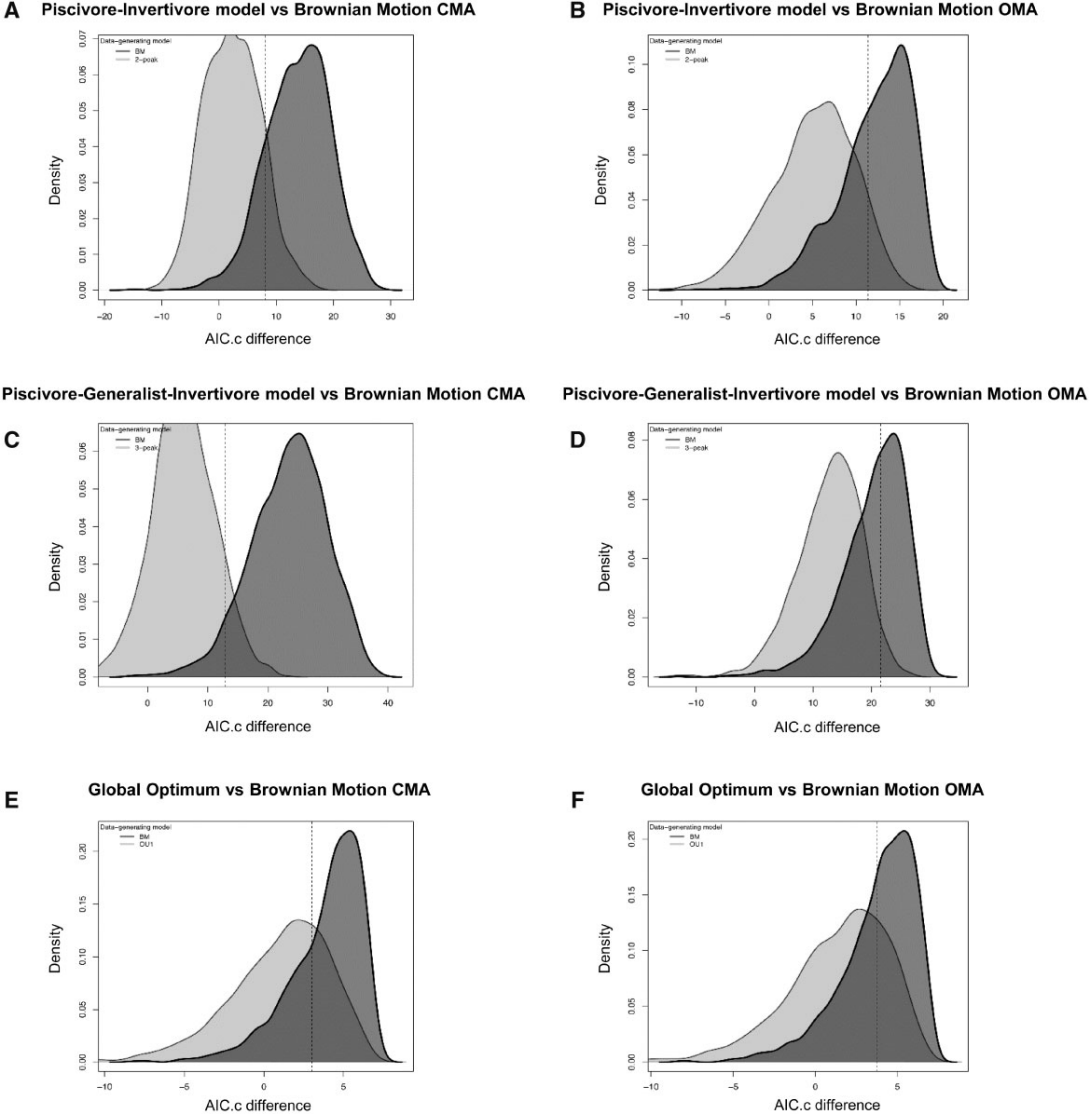
Results of parametric bootstrapping analysis showing simulated AICc differences between OU and BM models for closing (**A**, **C**, **E**) and opening (**B**, **D**, **F**) mechanical advantage. Dashed lines indicate observed AICc difference between OU and BM model.

When models (OU1, OU2, OU3, and BMM) of performance evolution are compared using *mvMORPH*, we initially find the highest support for a single-peak OU model for performance evolution (Supplementary Table S4). However, after quantifying the uncertainty in our parameter estimates using parametric bootstrapping, we find that we lack the statistical power to confidently distinguish between any of our models (Fig. 6).

## Discussion

### Relationships among morphology, performance, and ecology

The relationship among morphological traits and their associated ecologies has the potential to structure broad-scale of patterns among morphological evolution, habitat selection, and competition. In this study, we find that shape variance in the mandible region was the primary axis of variation in the 11 species sampled in this study. Furthermore, we find broad and phylogenetically structured patterns of trophic diversity within an assemblage of riverine-adapted Neotropical electric fishes whose diets range from invertivory to highly specialized forms of piscivory. We find that the evolution of skull shape and diet are generally congruent in this clade of fishes. Interestingly, we find that two functionally-defined sub-divisions of the skull closely track diet, with the neurocranium being more predictive (higher *r*^2^-value) than the mandible. It is possible that the mosaic of linkages and muscle attachments that functionally integrate the neurocranium and mandible have resulted in congruence between the shapes of the subdivisions and diet (Datovo and Vari 2013). The shape of mandible in particular has been found to closely track diet and is known to be highly plastic at early developmental stages in fishes (Hu and Albertson 2017). Furthermore, within gymnotiform fishes, the mandible has been shown to evolve nearly four times faster than its neighboring skull regions (Evans et al. 2019). It is therefore possible that these differences in trophic ecology are driving the elevated rates of shape evolution in the mandible within this clade of fishes.

Performance is often thought to function as the link between morphology and ecology. While performance is expected to closely track ecology, the relationship between morphology and performance may be less congruent as a result of many-to-one mapping. When we tested the relationship between mandible shape and performance, we found a significant relationship for CMA, and a non-significant relationship for OMA. These results suggest that the anterior region of the mandible is functionally more coupled to mechanical performance than is the posterior region of the mandible, the latter of which allows multiple morphologies to produce similar performance outputs. It is also possible that our phylogeny does not have enough transitions among states to be able to infer the true correlations or lack of correlations.

We recovered significant relationships between both measures of mechanical performance and TP. We found that species who feed at higher TPs exhibit lower mechanical advantages (i.e., mechanically faster jaws), while species that feed at lower TPs exhibit higher mechanical advantages (i.e., mechanically stronger jaws). This an intuitive result has been corroborated in other ecomorphological studies of the teleost mandible (Westneat 1994; Wainwright et al. 2004; Westneat 2005; Grubich et al. 2008). Mechanically fast jaws have been reported in several piscivorous fish species, and are thought to facilitate the capture of more elusive prey items. While mechanically strong jaws facilitate the consumption of hard bodied prey items.

When we modeled the adaptive landscape of performance, we were unable to successfully distinguish between models of performance evolution for two potential reasons: (1) we lacked that statistical power to adequately distinguish between models possibly due to our low sample size and lack of transitions between states and (2) the relationship between performance and TP may reflect more of a continuum than discrete differences. This can be seen in Fig. 5 where there is continuous variation along the performance axis.

### Lepidophagy in the river channels

We also quantify for the first time in gymnotiform fishes, the presence of lepidophagy. Lepidophagy is a highly specialized form of piscivory in which predators tear scales from the flanks and tails of prey fishes (Sazima 1983, 1988; Sazima and Machado 1990). This form of attack relies on high-speed ambush tactics and requires the ability to deliver quick but not necessarily strong bites to dislodge scales (Janovetz 2005; Martin and Wainwright 2013). Among Navajine fishes in this study, most of the species that incorporate fish tissues into their diets do so via lepidophagy. A recent study by Kolmann et al. (2018) showed that scale-eating piranhas and characins typically evolve faster closing jaws when compared with relatives who are trophic generalists. We recover a similar pattern in this study, where the generalist species *S. calhamazon* and *S. orthos* exhibit higher closing and OMAs than do their specialist lepidophagous relatives *S. raptor* and *S. duccis*.

## Conclusion

Interactions between form and function allow biologists to predict many broad scale patterns of macroevolution, habitat utilization, and competitive interactions within and among species. In this study we examine the relationship between morphology, performance, and trophic ecology in a clade of riverine-adapted electric fishes in the Amazon River. We recover significant relationships among morphology, ecology, and particular aspects of mechanical performance. We hypothesize that the shape of the skull and the performance of the mandible are being driven by strong selection to specialize on different trophic ecologies.

## Funding

This work was supported by United States National Science Foundation grants DEB 0614334, 0741450 and 1354511 to JSA, the Southern Regional Education Board Minority Doctoral Fellowship, and University of Minnesota, College of Agricultural and Natural Resource Sciences development funds, and the Brown University Presidential Postdoctoral Fellowship professional development fund to KME.

## Supplementary data

Supplementary data are available at IOB online.

## Supplementary Material

obz015_Supplementary_Data
