# Supplementary material for: Ecomorphology of Neotropical Electric Fishes: An Integrative Approach to Testing the Relationships between Form, Function, and Trophic Ecology
Source: Integr Org Biol. 2019 Jul 2;1(1):obz015. doi: 10.1093/iob/obz015 (PMC7671154; doi:10.1093/iob/obz015)
Supplement: obz015_Supplementary_Data [file obz015_supplementary_data.zip › Supplementary Methods.docx]

require(ape)

require(phytools)

require(geiger)

require(geomorph)

require(mvMORPH)

require(convevol)

require(ouch)

Gymn_tree<-read.tree("Full_Gymnotiform_tree.txt")

plot(Gymn_tree,cex=0.5)

Gymn_data <- read.csv("MA_3D.csv", header= T, row.names =1)

TreeOnly <- setdiff(Gymn_tree$tip.label,rownames(Gymn_data))

TreeOnly # Enter the name of the object we just created to see what's in it.

DataOnly <- setdiff(rownames(Gymn_data), Gymn_tree$tip.label)

DataOnly # Enter to see what species are in the data set but not the tree.

# In our case, we have overlap issues in both directions. Because we have data for fewer taxa than we have in our phylogeny, let's first prune our tree to just those species in the tree that were also measured before proceeding further.

# We'll prune the tree using drop.tip. We need to give it our tree, and a list of species to prune. We'll use the TreeOnly list of species names we just made to prune these species from the tree.

pruned_tree <- drop.tip(Gymn_tree,TreeOnly)

##############################

#Load Tree

##############################

phyloTime <- pruned_tree # Load a ultrametric tree

phyloTimeLadderized <- (ladderize(phyloTime)) # Ladderization

phyloTimeLadderized <- rescale(phyloTimeLadderized, "depth", 1) #This rescaling will make subsequent plotting functions somewhat easier. Even more importantly, it will often improve the performance of likelihood functions

plot(phyloTimeLadderized, cex=0.5, no.margin = T) #Plot ladderized and rescaled tree

add.scale.bar() # Add a simple scale bar indicating the scale for the branches in your tree

write.tree(phyloTimeLadderized, "phyloTimeLadderized.nwk")

phyloTimeLadderized <- read.tree("phyloTimeLadderized.nwk")

tree_2<-phyloTimeLadderized

#####################Full_Skull######################################

tmp_5<- read.csv("skull_full_data.csv", header= T, row.names =1,

stringsAsFactors = FALSE)

# here we are reading in a tab-delimited text file from MorphoJ, but this can be an issue with data from any outside program. The stringsAsFactors = FALSE is VERY important here

shape <- as.matrix(sapply(tmp_5[,-(1:3)], as.numeric))

# here we say, use all columns except the first three.

is.numeric(shape)

[1] TRUE # now it's numeric. Ready to go!

#Gonna Need names and Classifiers

names <- tmp_5[,1]

skull <- arrayspecs(shape[,1:ncol(shape)], 25,3)

#arrayspecs- substitute the column number where the coordinates begin for the 2, p=number of landmarks, k=number of dimensions

dimnames(skull)[[3]] <- names

################Full_Trophic_Data################

tp <- read.csv("MA.csv",header=TRUE, row.names =1)

tp_2<-tp$MeanTP

names(tp_2)<-row.names(tp)

####################Skull ANOVA#########################

gdf_sf_2 <- geomorph.data.frame(shape= skull, diet= tp_2)

anova_sf<-procD.lm(shape ~ diet, data = gdf_sf_2, iter = 999)

summary(anova_sf)

phy_PGLS_5<-procD.pgls(shape ~ diet, phy = tree_2, data = gdf_sf_2, iter = 999)

summary(phy_PGLS_5)

write.csv(phy_PGLS_5$aov.table, "full_skull_PGLS.csv")

######################Neurocranium###############

neuro<- read.csv("Full_neuro.csv", header= T, row.names =1,

stringsAsFactors = FALSE)

# here we are reading in a tab-delimited text file from MorphoJ, but this can be an issue with data from any outside program. The stringsAsFactors = FALSE is VERY important here

shape <- as.matrix(sapply(neuro[,-(1:2)], as.numeric))

# here we say, use all columns except the first three.

is.numeric(shape)

[1] TRUE # now it's numeric. Ready to go!

#Gonna Need names and Classifiers

names <- neuro[,1]

coords <- arrayspecs(shape[,1:ncol(shape)], 21,3)

#arrayspecs- substitute the column number where the coordinates begin for the 2, p=number of landmarks, k=number of dimensions

dimnames(coords)[[3]] <- names

###############Neuro Trophic ANOVA######################

gdf_neuro<- geomorph.data.frame(shape= coords, diet= tp_2)

anova_mf<-procD.lm(shape ~ diet, data = gdf_neuro, iter = 999)

summary(anova_mf)

phy_PGLS_6<-procD.pgls(shape ~ diet, phy = tree_2, data = gdf_neuro, iter = 999)

summary(phy_PGLS_6)

write.csv(phy_PGLS_6$aov.table, "full_neuro_PGLS.csv")

####################Mandible_Full_data###########

tmp_6 <- read.csv("Navajini_madible_full_data.csv", header= T, row.names =1,

stringsAsFactors = FALSE)

# here we are reading in a tab-delimited text file from MorphoJ, but this can be an issue with data from any outside program. The stringsAsFactors = FALSE is VERY important here

shape <- as.matrix(sapply(tmp_6[,-(1:3)], as.numeric))

# here we say, use all columns except the first three.

is.numeric(shape)

[1] TRUE # now it's numeric. Ready to go!

#Gonna Need names and Classifiers

names <- tmp_6[,1]

mandible <- arrayspecs(shape[,1:ncol(shape)], 4,3)

dimnames(mandible)[[3]] <- names

#################MA######################

MA<-read.csv("MA.csv", header=T, row.names=1)

C<- MA$CMA

names(C)<-row.names(MA)

O<- MA$OMA

names(O)<-row.names(MA)

####################ANOVA#########################

gdf_mf_2 <- geomorph.data.frame(shape= mandible, diet= tp_2,CMA=C, OMA=O)

anova_mf<-procD.lm(shape ~ diet, data = gdf_mf_2, iter = 999)

summary(anova_mf)

anova_CMA_S<-procD.lm(shape ~ CMA, data = gdf_mf_2, iter = 999)

summary(anova_CMA_S)

anova_OMA_S<-procD.lm(shape ~ OMA, data = gdf_mf_2, iter = 999)

summary(anova_OMA_S)

phy_PGLS_6<-procD.pgls(shape ~ diet, phy = tree_2, data = gdf_mf_2, iter = 999)

summary(phy_PGLS_6)

write.csv(phy_PGLS_6$aov.table, "full_mandible_PGLS.csv")

phy_PGLS_CMA<-procD.pgls(shape ~ CMA, phy = tree_2, data = gdf_mf_2, iter = 999)

summary(phy_PGLS_CMA)

phy_PGLS_OMA<-procD.pgls(shape ~ OMA, phy = tree_2, data = gdf_mf_2, iter = 999)

summary(phy_PGLS_OMA)

anova_mf<-procD.lm(shape ~ diet, data = gdf_mf_2, iter = 999)

###############################Mechanical Advantage###################

##########################Mechanical advantage ANOVA######################################

fit <- aov(CMA ~ MeanTP, data=MA)

summary(fit)

fit_2<- aov(OMA ~ MeanTP, data=MA)

summary(fit_2)

###########################MA VS. Mandible Shape###########

gdf_MCMA <- geomorph.data.frame(shape= mandible, CMA= CMA)

anova_MCMA<-procD.lm(shape ~ CMA, data = gdf_MCMA, iter = 999)

summary(anova_MCMA)

gdf_MOMA <- geomorph.data.frame(shape= mandible, OMA= OMA)

anova_MOMA<-procD.lm(shape ~ OMA, data = gdf_MOMA, iter = 999)

summary(anova_MOMA)

phy_PGLS_CMA<-procD.pgls(shape ~ CMA, phy = tree_2, data = gdf_MCMA, iter = 999)

summary(phy_PGLS_CMA)

phy_PGLS_OMA<-procD.pgls(shape ~ OMA, phy = tree_2, data = gdf_MOMA, iter = 999)

summary(phy_PGLS_OMA)

########################PGLS_Brownian########################################

tempTree <- tree_2

tempTree$edge.length <- tempTree$edge.length * 100 ########need to re-scale tree

data<-data.frame(CMA,OMA, tp_2, row.names=row.names(MA))

bm.nav<-corBrownian(phy=tempTree)

bm.gls_1<-gls(CMA~tp_2,correlation=bm.nav,data=data)

summary(bm.gls_1)

bm.gls_2<-gls(OMA~tp_2,correlation=bm.nav,data=data)

summary(bm.gls_2)

########################PGLS_OU############################################

ou.nav<-corMartins(1,phy=tempTree, fixed = TRUE)#########fixed alpha

OU.gls_1<-gls(CMA~tp_2,correlation=ou.nav,data=data)

summary(OU.gls_1)

OU.gls_2<-gls(OMA~tp_2,correlation=ou.nav,data=data)

summary(OU.gls_2)

#########Mechanical Advantage Integration#################

bm.gls_z<-gls(CMA~OMA,correlation=bm.nav,data=data)

summary(bm.gls_z)

OU.gls_Z<-gls(CMA~OMA,correlation=ou.nav,data=data)

summary(OU.gls_Z)

####################Phylogenetic Signal#######################

###############Trophic Position###############################

phylosig(tree_2,tp_2,method="K",test=TRUE)

##########CMA###################################

phylosig(tree_2,CMA,method="K",test=TRUE)

############OMA##############################################

phylosig(tree_2,OMA,method="K",test=TRUE)

#############Tukey_HSD#######

NI<-read.csv("Navajini isotopes.csv", row.names=1)

df<-data.frame(C=NI$C,N=NI$N, sp=NI$Species)

a1 <- aov(NI$N ~ NI$Species)

a2 <- aov(NI$C ~ NI$Species)

posthoc <- TukeyHSD(x=a1, 'NI$Species', conf.level=0.95)

posthoc_2 <- TukeyHSD(x=a2, 'NI$Species', conf.level=0.95)

#################MVMORPh###############

### DiscTraitOne

dataInput <- read.csv("peaks.csv") #Load discrete character data for Gymntotiformes

dataInput #Check out your data

workingData <- data.frame(dataInput[,2:5]) #Convert data in Data Frame in R

rownames(workingData) <- dataInput[,1]

attach(workingData)

name.check(phyloTimeLadderized, workingData)

head(workingData)

workingData <- workingData[phyloTimeLadderized$tip.label,] # sort our trait data to match the order of the tips in the tree

rownames(workingData) == phyloTimeLadderized$tip.label # check if it is sorted

#########peaks#########

discTraitone<- workingData[,2]###invertivore vs. generalist

names(discTraitone) <- rownames(workingData)

discTraittwo <- workingData[,3] # invertivore vs.generalist vs. piscivore

names(discTraittwo) <- rownames(workingData)

discTraitthree <- workingData[,4]## invertivore vs. piscivore

names(discTraitthree) <- rownames(workingData)

##############################

#AS_Recontruction - stochastic character map

##############################

trees_1<-make.simmap(phyloTimeLadderized,discTraitone,model="SYM", nsim=1000)

all.res_1 <- matrix(NA, nrow = 1000, ncol = 9)

for (i in 1:1000) try({

OU1<- mvOU(trees_1[[i]], cbind(C, O), model="OU1")

OUM<- mvOU(trees_1[[i]], cbind(C, O))

BM<- mvBM(trees_1[[i]], cbind(C, O))

all.res_1[i,][1] <- OU1$AICc

all.res_1[i,][2] <- OUM$AICc

all.res_1[i,][3] <- BM$AICc

all.res_1[i,][4] <- OU1$AIC

all.res_1[i,][5] <- OUM$AIC

all.res_1[i,][6] <- BM$AIC

all.res_1[i,][7] <- OU1$LogLik

all.res_1[i,][8] <- OUM$LogLik

all.res_1[i,][9] <- BM$LogLik

})

write.csv(all.res_1, file="mvmorph_res_mod_1.csv")

###Make Simmap#########

trees_2<-make.simmap(phyloTimeLadderized,discTraittwo,model="SYM", nsim=1000)

###################MVMORPHLOOP#############

all.res_2 <- matrix(NA, nrow = 1000, ncol = 9)

for (i in 1:1000) try({

OU1<- mvOU(trees_2[[i]], cbind(C, O), model="OU1")

OUM<- mvOU(trees_2[[i]], cbind(C, O))

BM<- mvBM(trees_2[[i]], cbind(C, O))

all.res_2[i,][1] <- OU1$AICc

all.res_2[i,][2] <- OUM$AICc

all.res_2[i,][3] <- BM$AICc

all.res_2[i,][4] <- OU1$AIC

all.res_2[i,][5] <- OUM$AIC

all.res_2[i,][6] <- BM$AIC

all.res_2[i,][7] <- OU1$LogLik

all.res_2[i,][8] <- OUM$LogLik

all.res_2[i,][9] <- BM$LogLik

})

write.csv(all.res_2, file="mvmorph_res_mod_2.csv")

###Make Simmap#########

trees_3<-make.simmap(phyloTimeLadderized,discTraitthree,model="SYM", nsim=1000)

###################MVMORPHLOOP#############

all.res_3 <- matrix(NA, nrow = 1000, ncol = 9)

for (i in 1:1000) try({

OU1<- mvOU(trees_3[[i]], cbind(C, O), model="OU1")

OUM<- mvOU(trees_3[[i]], cbind(C, O))

BM<- mvBM(trees_3[[i]], cbind(C, O))

all.res_3[i,][1] <- OU1$AICc

all.res_3[i,][2] <- OUM$AICc

all.res_3[i,][3] <- BM$AICc

all.res_3[i,][4] <- OU1$AIC

all.res_3[i,][5] <- OUM$AIC

all.res_3[i,][6] <- BM$AIC

all.res_3[i,][7] <- OU1$LogLik

all.res_3[i,][8] <- OUM$LogLik

all.res_3[i,][9] <- BM$LogLik

})

write.csv(all.res_3, file="mvmorph_res_mod_3.csv")

###############Neuro Trophic ANOVA######################

gdf_neuro<- geomorph.data.frame(shape= coords, diet= tp_2)

anova_mf<-procD.lm(shape ~ diet, data = gdf_neuro, iter = 999)

summary(anova_mf)

phy_PGLS_6<-procD.pgls(shape ~ diet, phy = tree_2, data = gdf_neuro, iter = 999)

summary(phy_PGLS_6)

write.csv(phy_PGLS_6$aov.table, "full_neuro_PGLS.csv")

############################3D Phylomrophospace################

X<- read.csv("Phylo3D.csv", header= T, row.names =1)

Y<- read.csv("MA.csv", header= T, row.names =1)

phylomorphospace3d(tree_2,Y, A=NULL,control=list(ftype='off'), angle=20,method="static")

tiff("ma_3d.tiff", width = 4, height = 4, units = 'in', res = 300)

####################Mechnical Advantage Plots############

MA<-read.csv("MA.csv", header=T, row.names = 1)

CMA<-ggplot(MA, aes(x=CMA, y=MeanTP)) +

geom_point()+

geom_smooth(method=lm)

######Remove Grid Lines#########

CMA + theme_bw() + theme(panel.border = element_blank(), panel.grid.major = element_blank(),

panel.grid.minor = element_blank(), axis.line = element_line(colour = "black"))

OMA<-ggplot(MA, aes(x=OMA, y=MeanTP)) +

geom_point()+

geom_smooth(method=lm)

######Remove Grid Lines#########

OMA + theme_bw() + theme(panel.border = element_blank(), panel.grid.major = element_blank(),

panel.grid.minor = element_blank(), axis.line = element_line(colour = "black"))

##############Continuous Character Map###############

dataInput <- read.csv("MA.csv") #Load character data for Gymntotiformes

dataInput #Check out your data

workingData <- data.frame(dataInput[,2:5]) #Convert data in Data Frame in R

rownames(workingData) <- dataInput[,1]

attach(workingData)

name.check(phyloTimeLadderized, workingData)

head(workingData)

workingData <- workingData[phyloTimeLadderized$tip.label,] # sort our trait data to match the order of the tips in the tree

rownames(workingData) == phyloTimeLadderized$tip.label # check if it is sorted

#####################Assesing convergence##############

Y<- read.csv("MA.csv", header= T, row.names =1)

convergent.sp <- rownames(Y)[which(Y$Guild == "G")]

################Invertivores#################

convergent.sp_2 <- rownames(Y)[which(Y$Guild == "I")]

CMA<- Y$CMA

OMA<-Y$OMA

names(CMA) <- names(OMA)<- rownames(Y)

################Number of time generalists have converged#####

res<-convnum(tree_2, cbind(CMA, OMA), convergent.sp)

###################Significance of Convergence

res_sig<-convnumsig(tree_2, cbind(CMA, OMA), convergent.sp,3000)

##############Invertivore Convergence#####################

res<-convnum(tree_2, cbind(CMA, OMA), convergent.sp_2)

###################Significance of Convergence

res_sig<-convnumsig(tree_2, cbind(CMA, OMA), convergent.sp_2,3000)

###############Using OUCH

ot<-ape2ouch(tree_2)

MA_b<-read.csv("MA_slim.csv",header=T, row.names=1)

sha<- as.data.frame(MA_b)

reg_1<-as.factor(MA_b[,4])

reg_2<-as.factor(MA_b[,2])

reg_1<-as.data.frame(reg_1)

reg_2<-as.data.frame(reg_2)

otd <- as(ot,"data.frame")

reg_1$labels<- rownames(MA_b)

sha$labels <- rownames(MA_b)

hmm<-Reduce(function(x, y) merge(x, y, all=TRUE), list(otd, sha, reg_1))

reg_1$labels<- rownames(MA_b)

reg_2$labels<- rownames(MA_b)

sha$labels <- rownames(MA_b)

otd <- merge(otd,sha,reg_1,reg_2,by="labels",all=TRUE)

rownames(hmm) <- hmm$nodes

print(hmm)

ot_x <- with(hmm,ouchtree(nodes=nodes,ancestors=ancestors,times=times,labels=labels))

b1 <- brown(tree=ot,data=otd[c("CMA","OMA")])

summary(b1)

tmp<- treedata(tree_2,MA_b)

#######################################################################################2-Peak parametric bootstrap############################################################

### You will probably have a tree in newick or nexus format.

### ouch expects a flat format - the easiest way

### is to get it into ape first and then use the ape2ouch function

### Here is an example - to generate a newick file and save to current working directory

require(ape)

require(phytools)

require(geiger)

require(geomorph)

require(mvMORPH)

require(convevol)

require(ouch)

Gymn_tree<-read.tree("Full_Gymnotiform_tree.txt")

plot(Gymn_tree,cex=0.5)

Gymn_data <- read.csv("MA_3D.csv", header= T, row.names =1)

TreeOnly <- setdiff(Gymn_tree$tip.label,rownames(Gymn_data))

TreeOnly # Enter the name of the object we just created to see what's in it.

DataOnly <- setdiff(rownames(Gymn_data), Gymn_tree$tip.label)

DataOnly # Enter to see what species are in the data set but not the tree.

# In our case, we have overlap issues in both directions. Because we have data for fewer taxa than we have in our phylogeny, let's first prune our tree to just those species in the tree that were also measured before proceeding further.

# We'll prune the tree using drop.tip. We need to give it our tree, and a list of species to prune. We'll use the TreeOnly list of species names we just made to prune these species from the tree.

pruned_tree <- drop.tip(Gymn_tree,TreeOnly)

##############################

#Load Tree

##############################

phyloTime <- pruned_tree # Load a ultrametric tree

phyloTimeLadderized <- (ladderize(phyloTime)) # Ladderization

phyloTimeLadderized <- rescale(phyloTimeLadderized, "depth", 1) #This rescaling will make subsequent plotting functions somewhat easier. Even more importantly, it will often improve the performance of likelihood functions

plot(phyloTimeLadderized, cex=0.5, no.margin = T) #Plot ladderized and rescaled tree

add.scale.bar() # Add a simple scale bar indicating the scale for the branches in your tree

write.tree(phyloTimeLadderized, "phyloTimeLadderized.nwk")

ape.tree <- read.tree("phyloTimeLadderized.nwk")

### Read your tree file into ape

## for nexus format use read.nexus() in ape

plot(ape.tree) ## to check that your tree is correct

tree <- ape2ouch(ape.tree) ## to get your tree into ouch format

### Print it to a file so you can open with a spreadsheet program and add your hypotheses

write.csv(as(tree, "data.frame"), file="MAtree.csv", row.names=F)

dat<-read.csv("MAtree.csv", header=T)

tree <- ouchtree(nodes=dat$nodes, ancestors=dat$ancestors, times=dat$times, labels=dat$labels)

plot(tree, node.names=T) ## show node numbers

dat <- as(tree, "data.frame") ## save tree as a data frame

### Hypotheses: you can add by using the paint function

### or by editing a spreadsheet by hand

pisc_inv<-paint(tree, subtree=c("2"="pisc","3"="inv","10"="inv"))

bm <- factor(rep("bm", each=length(pisc_inv))) ## only used for plotting

##dat$regimes <- as.factor("global")####Single Peak OU model

### To edit by hand,

### Open "owltree.csv" you will see that each node is on a row

### and is defined by the ancestor, descendant, and branchlength.

### You can add columns that will indicate hypotheses, etc.

# dat <- read.csv("owltree_hab.csv")

size <- c(0,0,0,0,0,0,0,0,0,0,0.303670805,0.229467186,0.343263132,0.365666765,0.518043566,0.329995505,0.409418116,0.384948505,0.458968238,0.413996601

,0.427677058)

OMA<- c(0,0,0,0,0,0,0,0,0,0,0.286554626,0.221207486,0.359906943,0.365990789,0.500316456,0.663001654,0.445875214,0.309796841,0.572238894,0.40957894,0.509851065

)

## simulate some size data

names(size)<- names(pisc_inv)<- names(bm)<- dat$nodes ## all objects must be named to keep track of species on rows

plot(tree, regimes=pisc_inv, lwd=5, frame.plot=F) ## plot habitat hypothesis

dat$pisc_inv<- pisc_inv

dat$size <- size

#######################################################################

############ OUCH analyses

## body size simulated with NO DIFFERENCE between habitats

## Note: This is a TINY tree. If the models donÊ»t converge try simulating

## another size dataset until it converges

#######################################################################

H <- list() ## list to hold model output

H$BM <- brown(size, tree)

H$pisc_inv <- hansen(size, tree, regimes =pisc_inv , sqrt.alpha=.5, sigma=1)

aicc.diff.obs<-summary(H$pisc_inv)$aic.c - summary(H$BM)$aic.c

# Calculate the observed

# aicc differences between more complex and less complex models

######## Model fit tables

fit <- t(sapply(H, function(x) {as.data.frame(rbind(unlist(summary(x)[c("dof", "loglik", "deviance", "aic", "aic.c", "sic")])))}))

print(dat)

print(fit, digits=3)

#############

### Now we generate simulated datasets under BM and habitat models

#############

bm.sim<-simulate(H$BM,nsim=2000) # 200 datasets based on fitted BM model

habitat.sim<-simulate(H$pisc_inv,nsim=2000) # should do 2000 for publication

#############

## Then we fit each simulation under each of the alternative models to

## to generate aicc, aic, sic differences

## So we have four sets of fits:

## A. BM fits when the data is generated under BM

## B. OUhabitat fits when data is generated under BM

## C. BM fits when data generated under OUhabitat

## D. OUhabitat fits when data generated under OUhabitat

##

## Use A B C D to generate these differences:

## aicc differences OU-BM generated under BM = B - A

## aicc differences OU-BM generated under OU = D - C

#############

#############

## BM - data generated on a BM model, specify # of simulations

## fit each simluation using each of the alternative models.

## You can also test different information criteria

#############

## BM sims fit to BM model

bmfits.bm.sim<-lapply(bm.sim, function(x) update(H$BM, data=x))# applies the model of interest to the simulated data

## get information criteria for each sim

bmaicc.bm<-sapply(bmfits.bm.sim, function(x) summary(x)$aic.c)# get aiccs

## BM sims fit to habitat model

habfits.bm.sim<-lapply(bm.sim, function(x) update(H$pisc_inv, data=x))

haicc.bm<-sapply(habfits.bm.sim, function(x) summary(x)$aic.c)

aicc.diffs.bm<-haicc.bm-bmaicc.bm # aicc differences habfit-BMfit when data

# is generated under BM model

#############

## Habitat fits

## For data generated under a habitat model, fit simluation using each of

## the alternative models.

#############

## Habitat sims fit to BM model

bmfits.hab.sim<-lapply(habitat.sim, function(x) update(H$BM, data=x))

bmaicc.hab<-sapply(bmfits.hab.sim, function(x) summary(x)$aic.c)

## Habitat sims fit to habitat model

habfits.hab.sim<-lapply(habitat.sim, function(x) update(H$pisc_inv, data=x))

haicc.hab<-sapply(habfits.hab.sim, function(x) summary(x)$aic.c)

aicc.diffs.hab<-haicc.hab-bmaicc.hab ## aicc difference between fits with hab model minus bm model when data is generated under hab model

maketransparent <- function( color, fraction) { # function to make colors transparent

col <- col2rgb(color)/255

col <- rgb(col[1], col[2], col[3], alpha = fraction)

return(col)

}

BMcol <- maketransparent("black", .5)

HABcol <- maketransparent("black", .2)

pdf(file="2_peaks.pdf")

par(oma=c(0,0,1,0))

#### AIC.c diffs ####

bmdens<-density(aicc.diffs.bm)

habdens<-density(aicc.diffs.hab)

obsdiff<- aicc.diff.obs

plot(bmdens, lwd=3, xlab="AIC.c difference", main = "")

lines(habdens, col=HABcol, lwd=3)

polygon(bmdens, col=BMcol)

polygon(habdens, col=HABcol)

abline(v= obsdiff, lty="dashed")

legend("topleft" , legend=c("BM", "2-peak"), xjust=0, col=c(BMcol, HABcol), lty=1, lwd=4, title="Data-generating model", cex=.75, bty="n")

legend("topright", legend="A", bty="n")

title("Piscivore-Invertivore model vs. Brownian Motion CMA", outer=TRUE, cex=1.5)

dev.off()

#######################################################################

## REPEAT:

## Simulate data with large size difference between habitats

## (generated under a HABITAT model with strong selection)

#######################################################################

dat$size <- c(0,0,0,0,0,0,0,0,0,0,0.303670805,0.229467186,0.343263132,0.365666765,0.518043566,0.329995505,0.409418116,0.384948505,0.458968238,0.413996601

,0.427677058) ## mean in forest =1, mean in grass=10

size <- dat$size

names(size) <- names(pisc_inv) <- dat$nodes ## all objects must be named to keep track of species on rows

############ OUCH analyses

H <- list() ## list to hold model output

H$BM <- brown(size, tree)

H$hab <- hansen(size, tree, pisc_inv, sqrt.alpha=.5, sigma=1)

aicc.diff.obs<-summary(H$hab)$aic.c - summary(H$BM)$aic.c

aic.diff.obs<-summary(H$hab)$aic - summary(H$BM)$aic

sic.diff.obs<-summary(H$hab)$sic - summary(H$BM)$sic

fit <- t(sapply(H, function(x) {as.data.frame(rbind(unlist(summary(x)[c("dof", "loglik", "deviance", "aic", "aic.c", "sic")])))}))

print(dat)

print(fit, digits=3) ## aicc is pretty wonky, probably tree too small

bm.sim<-simulate(H$BM,nsim=2000)

habitat.sim<-simulate(H$hab,nsim=2000)

bmfits.bm.sim<-lapply(bm.sim, function(x) update(H$BM, data=x))

bmaicc.bm<-sapply(bmfits.bm.sim, function(x) summary(x)$aic.c)

bmaic.bm<-sapply(bmfits.bm.sim, function(x) summary(x)$aic)

bmsic.bm<-sapply(bmfits.bm.sim, function(x) summary(x)$sic)

habfits.bm.sim<-lapply(bm.sim, function(x) update(H$hab, data=x))

haicc.bm<-sapply(habfits.bm.sim, function(x) summary(x)$aic.c)

haic.bm<-sapply(habfits.bm.sim, function(x) summary(x)$aic)

hsic.bm<-sapply(habfits.bm.sim, function(x) summary(x)$sic)

bmfits.hab.sim<-lapply(habitat.sim, function(x) update(H$BM, data=x))

bmaicc.hab<-sapply(bmfits.hab.sim, function(x) summary(x)$aic.c)

bmaic.hab<-sapply(bmfits.hab.sim, function(x) summary(x)$aic)

bmsic.hab<-sapply(bmfits.hab.sim, function(x) summary(x)$sic)

habfits.hab.sim<-lapply(habitat.sim, function(x) update(H$hab, data=x))

haicc.hab<-sapply(habfits.hab.sim, function(x) summary(x)$aic.c)

haic.hab<-sapply(habfits.hab.sim, function(x) summary(x)$aic)

hsic.hab<-sapply(habfits.hab.sim, function(x) summary(x)$sic)

aicc.diffs.bm<-haicc.bm-bmaicc.bm

aicc.diffs.hab<-haicc.hab-bmaicc.hab

aic.diffs.bm<-haic.bm-bmaic.bm

aic.diffs.hab<-haic.hab-bmaic.hab

sic.diffs.bm<-hsic.bm-bmsic.bm

sic.diffs.hab<-hsic.hab-bmsic.hab

############################################

## You can also calculate model selection frequencies

## simulated under the best-fit model

############################################

model.sel <- c(sum(aicc.diffs.hab<0), sum(aic.diffs.hab<0), sum(sic.diffs.hab<0))/length(aicc.diffs.hab)

model.sel <- data.frame(model.sel)

names(model.sel) <- "2-peak model"

row.names(model.sel) <- c("aicc", "aic", "sic")

print(model.sel)

pdf(file="Piscivore_Invertivore.pdf")

par(oma=c(0,0,1,0))

#### AIC.c diffs ####

bmdens<-density(aicc.diffs.bm)

habdens<-density(aicc.diffs.hab)

obsdiff<- aicc.diff.obs

plot(bmdens, lwd=3, xlab="AIC.c difference", main = "")

lines(habdens, col=HABcol, lwd=3)

polygon(bmdens, col=BMcol)

polygon(habdens, col=HABcol)

abline(v= obsdiff, lty="dashed")

legend(-80,.2 , legend=c("BM", "2-peak"), xjust=0, col=c(BMcol, HABcol), lty=1, lwd=4, title="Data-generating model", cex=.75, bty="n")

legend("topright", legend="A", bty="n")

title("BM vs. 2-peak", outer=TRUE, cex=1.5)

dev.off()

################################################################################################################

##OMA##

################################################################################################################

## simulate some size data

names(OMA)<- names(pisc_inv)<- names(bm)<- dat$nodes ## all objects must be named to keep track of species on rows

plot(tree, regimes=pisc_inv, lwd=5, frame.plot=F) ## plot habitat hypothesis

dat$pisc_inv<- pisc_inv

dat$OMA <- OMA

#######################################################################

############ OUCH analyses

## body size simulated with NO DIFFERENCE between habitats

## Note: This is a TINY tree. If the models donÊ»t converge try simulating

## another size dataset until it converges

#######################################################################

H <- list() ## list to hold model output

H$BM <- brown(OMA, tree)

H$pisc_inv <- hansen(OMA, tree, regimes =pisc_inv , sqrt.alpha=.5, sigma=1)

aicc.diff.obs<-summary(H$pisc_inv)$aic.c - summary(H$BM)$aic.c

# Calculate the observed

# aicc differences between more complex and less complex models

######## Model fit tables

fit <- t(sapply(H, function(x) {as.data.frame(rbind(unlist(summary(x)[c("dof", "loglik", "deviance", "aic", "aic.c", "sic")])))}))

print(dat)

print(fit, digits=3)

#############

### Now we generate simulated datasets under BM and habitat models

#############

bm.sim<-simulate(H$BM,nsim=2000) # 200 datasets based on fitted BM model

habitat.sim<-simulate(H$pisc_inv,nsim=2000) # should do 2000 for publication

#############

## Then we fit each simulation under each of the alternative models to

## to generate aicc, aic, sic differences

## So we have four sets of fits:

## A. BM fits when the data is generated under BM

## B. OUhabitat fits when data is generated under BM

## C. BM fits when data generated under OUhabitat

## D. OUhabitat fits when data generated under OUhabitat

##

## Use A B C D to generate these differences:

## aicc differences OU-BM generated under BM = B - A

## aicc differences OU-BM generated under OU = D - C

#############

#############

## BM - data generated on a BM model, specify # of simulations

## fit each simluation using each of the alternative models.

## You can also test different information criteria

#############

## BM sims fit to BM model

bmfits.bm.sim<-lapply(bm.sim, function(x) update(H$BM, data=x))# applies the model of interest to the simulated data

## get information criteria for each sim

bmaicc.bm<-sapply(bmfits.bm.sim, function(x) summary(x)$aic.c)# get aiccs

## BM sims fit to habitat model

habfits.bm.sim<-lapply(bm.sim, function(x) update(H$pisc_inv, data=x))

haicc.bm<-sapply(habfits.bm.sim, function(x) summary(x)$aic.c)

aicc.diffs.bm<-haicc.bm-bmaicc.bm # aicc differences habfit-BMfit when data

# is generated under BM model

#############

## Habitat fits

## For data generated under a habitat model, fit simluation using each of

## the alternative models.

#############

## Habitat sims fit to BM model

bmfits.hab.sim<-lapply(habitat.sim, function(x) update(H$BM, data=x))

bmaicc.hab<-sapply(bmfits.hab.sim, function(x) summary(x)$aic.c)

## Habitat sims fit to habitat model

habfits.hab.sim<-lapply(habitat.sim, function(x) update(H$pisc_inv, data=x))

haicc.hab<-sapply(habfits.hab.sim, function(x) summary(x)$aic.c)

aicc.diffs.hab<-haicc.hab-bmaicc.hab ## aicc difference between fits with hab model minus bm model when data is generated under hab model

maketransparent <- function( color, fraction) { # function to make colors transparent

col <- col2rgb(color)/255

col <- rgb(col[1], col[2], col[3], alpha = fraction)

return(col)

}

BMcol <- maketransparent("black", .5)

HABcol <- maketransparent("black", .2)

pdf(file="2_peaks_OMA.pdf")

par(oma=c(0,0,1,0))

#### AIC.c diffs ####

bmdens<-density(aicc.diffs.bm)

habdens<-density(aicc.diffs.hab)

obsdiff<- aicc.diff.obs

plot(bmdens, lwd=3, xlab="AIC.c difference", main = "")

lines(habdens, col=HABcol, lwd=3)

polygon(bmdens, col=BMcol)

polygon(habdens, col=HABcol)

abline(v= obsdiff, lty="dashed")

legend("topleft" , legend=c("BM", "2-peak"), xjust=0, col=c(BMcol, HABcol), lty=1, lwd=4, title="Data-generating model", cex=.75, bty="n")

legend("topright", legend="A", bty="n")

title("Piscivore-Invertivore model vs. Brownian Motion OMA", outer=TRUE, cex=1.5)

dev.off()

#######################################################################

## REPEAT:

## Simulate data with large size difference between habitats

## (generated under a HABITAT model with strong selection)

#######################################################################

dat$OMA <- c(0,0,0,0,0,0,0,0,0,0,0.286554626,0.221207486,0.359906943,0.365990789,0.500316456,0.663001654,0.445875214,0.309796841,0.572238894,0.40957894,0.509851065

) ## mean in forest =1, mean in grass=10

OMA <- dat$OMA

names(OMA) <- names(pisc_inv) <- dat$nodes ## all objects must be named to keep track of species on rows

############ OUCH analyses

H <- list() ## list to hold model output

H$BM <- brown(OMA, tree)

H$hab <- hansen(OMA, tree, pisc_inv, sqrt.alpha=.5, sigma=1)

aicc.diff.obs<-summary(H$hab)$aic.c - summary(H$BM)$aic.c

aic.diff.obs<-summary(H$hab)$aic - summary(H$BM)$aic

sic.diff.obs<-summary(H$hab)$sic - summary(H$BM)$sic

fit <- t(sapply(H, function(x) {as.data.frame(rbind(unlist(summary(x)[c("dof", "loglik", "deviance", "aic", "aic.c", "sic")])))}))

print(dat)

print(fit, digits=3) ## aicc is pretty wonky, probably tree too small

bm.sim<-simulate(H$BM,nsim=2000)

habitat.sim<-simulate(H$hab,nsim=2000)

bmfits.bm.sim<-lapply(bm.sim, function(x) update(H$BM, data=x))

bmaicc.bm<-sapply(bmfits.bm.sim, function(x) summary(x)$aic.c)

bmaic.bm<-sapply(bmfits.bm.sim, function(x) summary(x)$aic)

bmsic.bm<-sapply(bmfits.bm.sim, function(x) summary(x)$sic)

habfits.bm.sim<-lapply(bm.sim, function(x) update(H$hab, data=x))

haicc.bm<-sapply(habfits.bm.sim, function(x) summary(x)$aic.c)

haic.bm<-sapply(habfits.bm.sim, function(x) summary(x)$aic)

hsic.bm<-sapply(habfits.bm.sim, function(x) summary(x)$sic)

bmfits.hab.sim<-lapply(habitat.sim, function(x) update(H$BM, data=x))

bmaicc.hab<-sapply(bmfits.hab.sim, function(x) summary(x)$aic.c)

bmaic.hab<-sapply(bmfits.hab.sim, function(x) summary(x)$aic)

bmsic.hab<-sapply(bmfits.hab.sim, function(x) summary(x)$sic)

habfits.hab.sim<-lapply(habitat.sim, function(x) update(H$hab, data=x))

haicc.hab<-sapply(habfits.hab.sim, function(x) summary(x)$aic.c)

haic.hab<-sapply(habfits.hab.sim, function(x) summary(x)$aic)

hsic.hab<-sapply(habfits.hab.sim, function(x) summary(x)$sic)

aicc.diffs.bm<-haicc.bm-bmaicc.bm

aicc.diffs.hab<-haicc.hab-bmaicc.hab

aic.diffs.bm<-haic.bm-bmaic.bm

aic.diffs.hab<-haic.hab-bmaic.hab

sic.diffs.bm<-hsic.bm-bmsic.bm

sic.diffs.hab<-hsic.hab-bmsic.hab

############################################

## You can also calculate model selection frequencies

## simulated under the best-fit model

############################################

model.sel <- c(sum(aicc.diffs.hab<0), sum(aic.diffs.hab<0), sum(sic.diffs.hab<0))/length(aicc.diffs.hab)

model.sel <- data.frame(model.sel)

names(model.sel) <- "2-peak model"

row.names(model.sel) <- c("aicc", "aic", "sic")

print(model.sel)

pdf(file="Piscivore_Invertivore_OMA.pdf")

par(oma=c(0,0,1,0))

#### AIC.c diffs ####

bmdens<-density(aicc.diffs.bm)

habdens<-density(aicc.diffs.hab)

obsdiff<- aicc.diff.obs

plot(bmdens, lwd=3, xlab="AIC.c difference", main = "")

lines(habdens, col=HABcol, lwd=3)

polygon(bmdens, col=BMcol)

polygon(habdens, col=HABcol)

abline(v= obsdiff, lty="dashed")

legend(-80,.2 , legend=c("BM", "2-peak"), xjust=0, col=c(BMcol, HABcol), lty=1, lwd=4, title="Data-generating model", cex=.75, bty="n")

legend("topright", legend="A", bty="n")

title("BM vs. 2-peak", outer=TRUE, cex=1.5)

dev.off()

#######################################################################################3-Peak parametric bootstrap############################################################

### You will probably have a tree in newick or nexus format.

### ouch expects a flat format - the easiest way

### is to get it into ape first and then use the ape2ouch function

### Here is an example - to generate a newick file and save to current working directory

require(ape)

require(phytools)

require(geiger)

require(geomorph)

require(mvMORPH)

require(convevol)

require(ouch)

Gymn_tree<-read.tree("Full_Gymnotiform_tree.txt")

plot(Gymn_tree,cex=0.5)

Gymn_data <- read.csv("MA_3D.csv", header= T, row.names =1)

TreeOnly <- setdiff(Gymn_tree$tip.label,rownames(Gymn_data))

TreeOnly # Enter the name of the object we just created to see what's in it.

DataOnly <- setdiff(rownames(Gymn_data), Gymn_tree$tip.label)

DataOnly # Enter to see what species are in the data set but not the tree.

# In our case, we have overlap issues in both directions. Because we have data for fewer taxa than we have in our phylogeny, let's first prune our tree to just those species in the tree that were also measured before proceeding further.

# We'll prune the tree using drop.tip. We need to give it our tree, and a list of species to prune. We'll use the TreeOnly list of species names we just made to prune these species from the tree.

pruned_tree <- drop.tip(Gymn_tree,TreeOnly)

##############################

#Load Tree

##############################

phyloTime <- pruned_tree # Load a ultrametric tree

phyloTimeLadderized <- (ladderize(phyloTime)) # Ladderization

phyloTimeLadderized <- rescale(phyloTimeLadderized, "depth", 1) #This rescaling will make subsequent plotting functions somewhat easier. Even more importantly, it will often improve the performance of likelihood functions

plot(phyloTimeLadderized, cex=0.5, no.margin = T) #Plot ladderized and rescaled tree

add.scale.bar() # Add a simple scale bar indicating the scale for the branches in your tree

write.tree(phyloTimeLadderized, "phyloTimeLadderized.nwk")

ape.tree <- read.tree("phyloTimeLadderized.nwk")

### Read your tree file into ape

## for nexus format use read.nexus() in ape

plot(ape.tree) ## to check that your tree is correct

tree <- ape2ouch(ape.tree) ## to get your tree into ouch format

### Print it to a file so you can open with a spreadsheet program and add your hypotheses

write.csv(as(tree, "data.frame"), file="MAtree.csv", row.names=F)

dat<-read.csv("MAtree.csv", header=T)

tree <- ouchtree(nodes=dat$nodes, ancestors=dat$ancestors, times=dat$times, labels=dat$labels)

plot(tree, node.names=T) ## show node numbers

dat <- as(tree, "data.frame") ## save tree as a data frame

### Hypotheses: you can add by using the paint function

### or by editing a spreadsheet by hand

gen_pisc_inv<-paint(tree, subtree=c("3"="gen","6"="inv", "8"="inv", "2"="pis"), branch=c("16"="gen", "18"="gen", "20"="gen"))

bm <- factor(rep("bm", each=length(gen_pisc_inv))) ## only used for plotting

##dat$regimes <- as.factor("global")####Single Peak OU model

### To edit by hand,

### Open "owltree.csv" you will see that each node is on a row

### and is defined by the ancestor, descendant, and branchlength.

### You can add columns that will indicate hypotheses, etc.

# dat <- read.csv("owltree_hab.csv")

size <- c(0,0,0,0,0,0,0,0,0,0,0.303670805,0.229467186,0.343263132,0.365666765,0.518043566,0.329995505,0.409418116,0.384948505,0.458968238,0.413996601

,0.427677058)

OMA<- c(0,0,0,0,0,0,0,0,0,0,0.286554626,0.221207486,0.359906943,0.365990789,0.500316456,0.663001654,0.445875214,0.309796841,0.572238894,0.40957894,0.509851065

)

## simulate some size data

names(size)<- names(gen_pisc_inv)<- names(bm)<- dat$nodes ## all objects must be named to keep track of species on rows

plot(tree, regimes=gen_pisc_inv, lwd=5, frame.plot=F) ## plot habitat hypothesis

dat$gen_pisc_inv<- gen_pisc_inv

dat$size <- size

#######################################################################

############ OUCH analyses

## body size simulated with NO DIFFERENCE between habitats

## Note: This is a TINY tree. If the models donÊ»t converge try simulating

## another size dataset until it converges

#######################################################################

H <- list() ## list to hold model output

H$BM <- brown(size, tree)

H$gen_pisc_inv <- hansen(size, tree, regimes =gen_pisc_inv , sqrt.alpha=.5, sigma=1)

aicc.diff.obs<-summary(H$gen_pisc_inv)$aic.c - summary(H$BM)$aic.c

# Calculate the observed

# aicc differences between more complex and less complex models

######## Model fit tables

fit <- t(sapply(H, function(x) {as.data.frame(rbind(unlist(summary(x)[c("dof", "loglik", "deviance", "aic", "aic.c", "sic")])))}))

print(dat)

print(fit, digits=3)

#############

### Now we generate simulated datasets under BM and habitat models

#############

bm.sim<-simulate(H$BM,nsim=2000) # 200 datasets based on fitted BM model

habitat.sim<-simulate(H$gen_pisc_inv,nsim=2000) # should do 2000 for publication

#############

## Then we fit each simulation under each of the alternative models to

## to generate aicc, aic, sic differences

## So we have four sets of fits:

## A. BM fits when the data is generated under BM

## B. OUhabitat fits when data is generated under BM

## C. BM fits when data generated under OUhabitat

## D. OUhabitat fits when data generated under OUhabitat

##

## Use A B C D to generate these differences:

## aicc differences OU-BM generated under BM = B - A

## aicc differences OU-BM generated under OU = D - C

#############

#############

## BM - data generated on a BM model, specify # of simulations

## fit each simluation using each of the alternative models.

## You can also test different information criteria

#############

## BM sims fit to BM model

bmfits.bm.sim<-lapply(bm.sim, function(x) update(H$BM, data=x))# applies the model of interest to the simulated data

## get information criteria for each sim

bmaicc.bm<-sapply(bmfits.bm.sim, function(x) summary(x)$aic.c)# get aiccs

## BM sims fit to habitat model

habfits.bm.sim<-lapply(bm.sim, function(x) update(H$gen_pisc_inv, data=x))

haicc.bm<-sapply(habfits.bm.sim, function(x) summary(x)$aic.c)

aicc.diffs.bm<-haicc.bm-bmaicc.bm # aicc differences habfit-BMfit when data

# is generated under BM model

#############

## Habitat fits

## For data generated under a habitat model, fit simluation using each of

## the alternative models.

#############

## Habitat sims fit to BM model

bmfits.hab.sim<-lapply(habitat.sim, function(x) update(H$BM, data=x))

bmaicc.hab<-sapply(bmfits.hab.sim, function(x) summary(x)$aic.c)

## Habitat sims fit to habitat model

habfits.hab.sim<-lapply(habitat.sim, function(x) update(H$gen_pisc_inv, data=x))

haicc.hab<-sapply(habfits.hab.sim, function(x) summary(x)$aic.c)

aicc.diffs.hab<-haicc.hab-bmaicc.hab ## aicc difference between fits with hab model minus bm model when data is generated under hab model

maketransparent <- function( color, fraction) { # function to make colors transparent

col <- col2rgb(color)/255

col <- rgb(col[1], col[2], col[3], alpha = fraction)

return(col)

}

BMcol <- maketransparent("black", .5)

HABcol <- maketransparent("black", .2)

pdf(file="3_peaks.pdf")

par(oma=c(0,0,1,0))

#### AIC.c diffs ####

bmdens<-density(aicc.diffs.bm)

habdens<-density(aicc.diffs.hab)

obsdiff<- aicc.diff.obs

plot(bmdens, lwd=3, xlab="AIC.c difference", main = "")

lines(habdens, col=HABcol, lwd=3)

polygon(bmdens, col=BMcol)

polygon(habdens, col=HABcol)

abline(v= obsdiff, lty="dashed")

legend("topleft" , legend=c("BM", "3-peak"), xjust=0, col=c(BMcol, HABcol), lty=1, lwd=4, title="Data-generating model", cex=.75, bty="n")

legend("topright", legend="A", bty="n")

title("Piscivore-Generalist-Invertivore model vs. Brownian Motion CMA", outer=TRUE, cex=1.5)

dev.off()

#######################################################################

## REPEAT:

## Simulate data with large size difference between habitats

## (generated under a HABITAT model with strong selection)

#######################################################################

dat$size <- c(0,0,0,0,0,0,0,0,0,0,0.303670805,0.229467186,0.343263132,0.365666765,0.518043566,0.329995505,0.409418116,0.384948505,0.458968238,0.413996601

,0.427677058) ## mean in forest =1, mean in grass=10

size <- dat$size

names(size) <- names(gen_pisc_inv) <- dat$nodes ## all objects must be named to keep track of species on rows

############ OUCH analyses

H <- list() ## list to hold model output

H$BM <- brown(size, tree)

H$hab <- hansen(size, tree, gen_pisc_inv, sqrt.alpha=.5, sigma=1)

aicc.diff.obs<-summary(H$hab)$aic.c - summary(H$BM)$aic.c

aic.diff.obs<-summary(H$hab)$aic - summary(H$BM)$aic

sic.diff.obs<-summary(H$hab)$sic - summary(H$BM)$sic

fit <- t(sapply(H, function(x) {as.data.frame(rbind(unlist(summary(x)[c("dof", "loglik", "deviance", "aic", "aic.c", "sic")])))}))

print(dat)

print(fit, digits=3) ## aicc is pretty wonky, probably tree too small

bm.sim<-simulate(H$BM,nsim=2000)

habitat.sim<-simulate(H$hab,nsim=2000)

bmfits.bm.sim<-lapply(bm.sim, function(x) update(H$BM, data=x))

bmaicc.bm<-sapply(bmfits.bm.sim, function(x) summary(x)$aic.c)

bmaic.bm<-sapply(bmfits.bm.sim, function(x) summary(x)$aic)

bmsic.bm<-sapply(bmfits.bm.sim, function(x) summary(x)$sic)

habfits.bm.sim<-lapply(bm.sim, function(x) update(H$hab, data=x))

haicc.bm<-sapply(habfits.bm.sim, function(x) summary(x)$aic.c)

haic.bm<-sapply(habfits.bm.sim, function(x) summary(x)$aic)

hsic.bm<-sapply(habfits.bm.sim, function(x) summary(x)$sic)

bmfits.hab.sim<-lapply(habitat.sim, function(x) update(H$BM, data=x))

bmaicc.hab<-sapply(bmfits.hab.sim, function(x) summary(x)$aic.c)

bmaic.hab<-sapply(bmfits.hab.sim, function(x) summary(x)$aic)

bmsic.hab<-sapply(bmfits.hab.sim, function(x) summary(x)$sic)

habfits.hab.sim<-lapply(habitat.sim, function(x) update(H$hab, data=x))

haicc.hab<-sapply(habfits.hab.sim, function(x) summary(x)$aic.c)

haic.hab<-sapply(habfits.hab.sim, function(x) summary(x)$aic)

hsic.hab<-sapply(habfits.hab.sim, function(x) summary(x)$sic)

aicc.diffs.bm<-haicc.bm-bmaicc.bm

aicc.diffs.hab<-haicc.hab-bmaicc.hab

aic.diffs.bm<-haic.bm-bmaic.bm

aic.diffs.hab<-haic.hab-bmaic.hab

sic.diffs.bm<-hsic.bm-bmsic.bm

sic.diffs.hab<-hsic.hab-bmsic.hab

############################################

## You can also calculate model selection frequencies

## simulated under the best-fit model

############################################

model.sel <- c(sum(aicc.diffs.hab<0), sum(aic.diffs.hab<0), sum(sic.diffs.hab<0))/length(aicc.diffs.hab)

model.sel <- data.frame(model.sel)

names(model.sel) <- "3-peak model"

row.names(model.sel) <- c("aicc", "aic", "sic")

print(model.sel)

pdf(file="Piscivore_Generalist_Invertivore.pdf")

par(oma=c(0,0,1,0))

#### AIC.c diffs ####

bmdens<-density(aicc.diffs.bm)

habdens<-density(aicc.diffs.hab)

obsdiff<- aicc.diff.obs

plot(bmdens, lwd=3, xlab="AIC.c difference", main = "")

lines(habdens, col=HABcol, lwd=3)

polygon(bmdens, col=BMcol)

polygon(habdens, col=HABcol)

abline(v= obsdiff, lty="dashed")

legend(-80,.2 , legend=c("BM", "3-peak"), xjust=0, col=c(BMcol, HABcol), lty=1, lwd=4, title="Data-generating model", cex=.75, bty="n")

legend("topright", legend="A", bty="n")

title("BM vs. 3-peak", outer=TRUE, cex=1.5)

dev.off()

################################################################################################################

##OMA##

################################################################################################################

## simulate some size data

names(OMA)<- names(gen_pisc_inv)<- names(bm)<- dat$nodes ## all objects must be named to keep track of species on rows

plot(tree, regimes=gen_pisc_inv, lwd=5, frame.plot=F) ## plot habitat hypothesis

dat$gen_pisc_inv<- gen_pisc_inv

dat$OMA <- OMA

#######################################################################

############ OUCH analyses

## body size simulated with NO DIFFERENCE between habitats

## Note: This is a TINY tree. If the models donÊ»t converge try simulating

## another size dataset until it converges

#######################################################################

H <- list() ## list to hold model output

H$BM <- brown(OMA, tree)

H$gen_pisc_inv <- hansen(OMA, tree, regimes =gen_pisc_inv , sqrt.alpha=.5, sigma=1)

aicc.diff.obs<-summary(H$gen_pisc_inv)$aic.c - summary(H$BM)$aic.c

# Calculate the observed

# aicc differences between more complex and less complex models

######## Model fit tables

fit <- t(sapply(H, function(x) {as.data.frame(rbind(unlist(summary(x)[c("dof", "loglik", "deviance", "aic", "aic.c", "sic")])))}))

print(dat)

print(fit, digits=3)

#############

### Now we generate simulated datasets under BM and habitat models

#############

bm.sim<-simulate(H$BM,nsim=2000) # 200 datasets based on fitted BM model

habitat.sim<-simulate(H$gen_pisc_inv,nsim=2000) # should do 2000 for publication

#############

## Then we fit each simulation under each of the alternative models to

## to generate aicc, aic, sic differences

## So we have four sets of fits:

## A. BM fits when the data is generated under BM

## B. OUhabitat fits when data is generated under BM

## C. BM fits when data generated under OUhabitat

## D. OUhabitat fits when data generated under OUhabitat

##

## Use A B C D to generate these differences:

## aicc differences OU-BM generated under BM = B - A

## aicc differences OU-BM generated under OU = D - C

#############

#############

## BM - data generated on a BM model, specify # of simulations

## fit each simluation using each of the alternative models.

## You can also test different information criteria

#############

## BM sims fit to BM model

bmfits.bm.sim<-lapply(bm.sim, function(x) update(H$BM, data=x))# applies the model of interest to the simulated data

## get information criteria for each sim

bmaicc.bm<-sapply(bmfits.bm.sim, function(x) summary(x)$aic.c)# get aiccs

## BM sims fit to habitat model

habfits.bm.sim<-lapply(bm.sim, function(x) update(H$gen_pisc_inv, data=x))

haicc.bm<-sapply(habfits.bm.sim, function(x) summary(x)$aic.c)

aicc.diffs.bm<-haicc.bm-bmaicc.bm # aicc differences habfit-BMfit when data

# is generated under BM model

#############

## Habitat fits

## For data generated under a habitat model, fit simluation using each of

## the alternative models.

#############

## Habitat sims fit to BM model

bmfits.hab.sim<-lapply(habitat.sim, function(x) update(H$BM, data=x))

bmaicc.hab<-sapply(bmfits.hab.sim, function(x) summary(x)$aic.c)

## Habitat sims fit to habitat model

habfits.hab.sim<-lapply(habitat.sim, function(x) update(H$gen_pisc_inv, data=x))

haicc.hab<-sapply(habfits.hab.sim, function(x) summary(x)$aic.c)

aicc.diffs.hab<-haicc.hab-bmaicc.hab ## aicc difference between fits with hab model minus bm model when data is generated under hab model

maketransparent <- function( color, fraction) { # function to make colors transparent

col <- col2rgb(color)/255

col <- rgb(col[1], col[2], col[3], alpha = fraction)

return(col)

}

BMcol <- maketransparent("black", .5)

HABcol <- maketransparent("black", .2)

pdf(file="3_peaks_OMA.pdf")

par(oma=c(0,0,1,0))

#### AIC.c diffs ####

bmdens<-density(aicc.diffs.bm)

habdens<-density(aicc.diffs.hab)

obsdiff<- aicc.diff.obs

plot(bmdens, lwd=3, xlab="AIC.c difference", main = "")

lines(habdens, col=HABcol, lwd=3)

polygon(bmdens, col=BMcol)

polygon(habdens, col=HABcol)

abline(v= obsdiff, lty="dashed")

legend("topleft" , legend=c("BM", "3-peak"), xjust=0, col=c(BMcol, HABcol), lty=1, lwd=4, title="Data-generating model", cex=.75, bty="n")

legend("topright", legend="A", bty="n")

title("Piscivore-Generalist-Invertivore model vs. Brownian Motion OMA", outer=TRUE, cex=1.5)

dev.off()

#######################################################################

## REPEAT:

## Simulate data with large size difference between habitats

## (generated under a HABITAT model with strong selection)

#######################################################################

dat$OMA <- c(0,0,0,0,0,0,0,0,0,0,0.286554626,0.221207486,0.359906943,0.365990789,0.500316456,0.663001654,0.445875214,0.309796841,0.572238894,0.40957894,0.509851065

) ## mean in forest =1, mean in grass=10

OMA <- dat$OMA

names(OMA) <- names(gen_pisc_inv) <- dat$nodes ## all objects must be named to keep track of species on rows

############ OUCH analyses

H <- list() ## list to hold model output

H$BM <- brown(OMA, tree)

H$hab <- hansen(OMA, tree, gen_pisc_inv, sqrt.alpha=.5, sigma=1)

aicc.diff.obs<-summary(H$hab)$aic.c - summary(H$BM)$aic.c

aic.diff.obs<-summary(H$hab)$aic - summary(H$BM)$aic

sic.diff.obs<-summary(H$hab)$sic - summary(H$BM)$sic

fit <- t(sapply(H, function(x) {as.data.frame(rbind(unlist(summary(x)[c("dof", "loglik", "deviance", "aic", "aic.c", "sic")])))}))

print(dat)

print(fit, digits=3) ## aicc is pretty wonky, probably tree too small

bm.sim<-simulate(H$BM,nsim=2000)

habitat.sim<-simulate(H$hab,nsim=2000)

bmfits.bm.sim<-lapply(bm.sim, function(x) update(H$BM, data=x))

bmaicc.bm<-sapply(bmfits.bm.sim, function(x) summary(x)$aic.c)

bmaic.bm<-sapply(bmfits.bm.sim, function(x) summary(x)$aic)

bmsic.bm<-sapply(bmfits.bm.sim, function(x) summary(x)$sic)

habfits.bm.sim<-lapply(bm.sim, function(x) update(H$hab, data=x))

haicc.bm<-sapply(habfits.bm.sim, function(x) summary(x)$aic.c)

haic.bm<-sapply(habfits.bm.sim, function(x) summary(x)$aic)

hsic.bm<-sapply(habfits.bm.sim, function(x) summary(x)$sic)

bmfits.hab.sim<-lapply(habitat.sim, function(x) update(H$BM, data=x))

bmaicc.hab<-sapply(bmfits.hab.sim, function(x) summary(x)$aic.c)

bmaic.hab<-sapply(bmfits.hab.sim, function(x) summary(x)$aic)

bmsic.hab<-sapply(bmfits.hab.sim, function(x) summary(x)$sic)

habfits.hab.sim<-lapply(habitat.sim, function(x) update(H$hab, data=x))

haicc.hab<-sapply(habfits.hab.sim, function(x) summary(x)$aic.c)

haic.hab<-sapply(habfits.hab.sim, function(x) summary(x)$aic)

hsic.hab<-sapply(habfits.hab.sim, function(x) summary(x)$sic)

aicc.diffs.bm<-haicc.bm-bmaicc.bm

aicc.diffs.hab<-haicc.hab-bmaicc.hab

aic.diffs.bm<-haic.bm-bmaic.bm

aic.diffs.hab<-haic.hab-bmaic.hab

sic.diffs.bm<-hsic.bm-bmsic.bm

sic.diffs.hab<-hsic.hab-bmsic.hab

############################################

## You can also calculate model selection frequencies

## simulated under the best-fit model

############################################

model.sel <- c(sum(aicc.diffs.hab<0), sum(aic.diffs.hab<0), sum(sic.diffs.hab<0))/length(aicc.diffs.hab)

model.sel <- data.frame(model.sel)

names(model.sel) <- "3-peak model"

row.names(model.sel) <- c("aicc", "aic", "sic")

print(model.sel)

pdf(file="Piscivore_Generalist_Invertivore_OMA.pdf")

par(oma=c(0,0,1,0))

#### AIC.c diffs ####

bmdens<-density(aicc.diffs.bm)

habdens<-density(aicc.diffs.hab)

obsdiff<- aicc.diff.obs

plot(bmdens, lwd=3, xlab="AIC.c difference", main = "")

lines(habdens, col=HABcol, lwd=3)

polygon(bmdens, col=BMcol)

polygon(habdens, col=HABcol)

abline(v= obsdiff, lty="dashed")

legend(-80,.2 , legend=c("BM", "2-peak"), xjust=0, col=c(BMcol, HABcol), lty=1, lwd=4, title="Data-generating model", cex=.75, bty="n")

legend("topright", legend="A", bty="n")

title("BM vs. 2-peak", outer=TRUE, cex=1.5)

dev.off()

#######################################################################################OU1 parametric bootstrap############################################################

### You will probably have a tree in newick or nexus format.

### ouch expects a flat format - the easiest way

### is to get it into ape first and then use the ape2ouch function

### Here is an example - to generate a newick file and save to current working directory

require(ape)

require(phytools)

require(geiger)

require(geomorph)

require(mvMORPH)

require(convevol)

require(ouch)

Gymn_tree<-read.tree("Full_Gymnotiform_tree.txt")

plot(Gymn_tree,cex=0.5)

Gymn_data <- read.csv("MA_3D.csv", header= T, row.names =1)

TreeOnly <- setdiff(Gymn_tree$tip.label,rownames(Gymn_data))

TreeOnly # Enter the name of the object we just created to see what's in it.

DataOnly <- setdiff(rownames(Gymn_data), Gymn_tree$tip.label)

DataOnly # Enter to see what species are in the data set but not the tree.

# In our case, we have overlap issues in both directions. Because we have data for fewer taxa than we have in our phylogeny, let's first prune our tree to just those species in the tree that were also measured before proceeding further.

# We'll prune the tree using drop.tip. We need to give it our tree, and a list of species to prune. We'll use the TreeOnly list of species names we just made to prune these species from the tree.

pruned_tree <- drop.tip(Gymn_tree,TreeOnly)

##############################

#Load Tree

##############################

phyloTime <- pruned_tree # Load a ultrametric tree

phyloTimeLadderized <- (ladderize(phyloTime)) # Ladderization

phyloTimeLadderized <- rescale(phyloTimeLadderized, "depth", 1) #This rescaling will make subsequent plotting functions somewhat easier. Even more importantly, it will often improve the performance of likelihood functions

plot(phyloTimeLadderized, cex=0.5, no.margin = T) #Plot ladderized and rescaled tree

add.scale.bar() # Add a simple scale bar indicating the scale for the branches in your tree

write.tree(phyloTimeLadderized, "phyloTimeLadderized.nwk")

ape.tree <- read.tree("phyloTimeLadderized.nwk")

### Read your tree file into ape

## for nexus format use read.nexus() in ape

plot(ape.tree) ## to check that your tree is correct

tree <- ape2ouch(ape.tree) ## to get your tree into ouch format

### Print it to a file so you can open with a spreadsheet program and add your hypotheses

write.csv(as(tree, "data.frame"), file="MAtree.csv", row.names=F)

dat<-read.csv("MAtree.csv", header=T)

tree <- ouchtree(nodes=dat$nodes, ancestors=dat$ancestors, times=dat$times, labels=dat$labels)

plot(tree, node.names=T) ## show node numbers

dat <- as(tree, "data.frame") ## save tree as a data frame

### Hypotheses: you can add by using the paint function

### or by editing a spreadsheet by hand

#gen_pisc_inv<-paint(tree, subtree=c("3"="gen","6"="inv", "8"="inv", "2"="pis"), branch=c("16"="gen", "18"="gen", "20"="gen"))

#gen_inv<-paint(tree, subtree=c("3"="gen","6"="inv", "8"="inv", "2"="gen"), branch=c("16"="gen", "18"="gen", "20"="gen"))

#pisc_inv<-paint(tree, subtree=c("2"="pisc","3"="inv","10"="inv"))

#clade<-paint(tree, subtree=c("4"="stern","10"="inv"))

## only used for plotting

dat$regimes <- as.factor("global")####Single Peak OU model

bm <- factor(rep("bm", each=length(dat$regimes)))## only used for plotting

### To edit by hand,

### Open "owltree.csv" you will see that each node is on a row

### and is defined by the ancestor, descendant, and branchlength.

### You can add columns that will indicate hypotheses, etc.

# dat <- read.csv("owltree_hab.csv")

size <- c(0,0,0,0,0,0,0,0,0,0,0.303670805,0.229467186,0.343263132,0.365666765,0.518043566,0.329995505,0.409418116,0.384948505,0.458968238,0.413996601

,0.427677058)

OMA<- c(0,0,0,0,0,0,0,0,0,0,0.286554626,0.221207486,0.359906943,0.365990789,0.500316456,0.663001654,0.445875214,0.309796841,0.572238894,0.40957894,0.509851065

)

## simulate some size data

names(size)<- names(dat$regimes)<- names(bm)<- dat$nodes ## all objects must be named to keep track of species on rows

plot(tree, regimes=dat$regimes, lwd=5, frame.plot=F) ## plot habitat hypothesis

dat$gen_pisc_inv<- gen_pisc_inv

dat$size <- size

#######################################################################

############ OUCH analyses

## body size simulated with NO DIFFERENCE between habitats

## Note: This is a TINY tree. If the models donÊ»t converge try simulating

## another size dataset until it converges

#######################################################################

H <- list() ## list to hold model output

H$BM <- brown(size, tree)

H$OU1 <- hansen(size, tree, regimes =dat["regimes"] , sqrt.alpha=.5, sigma=1)

aicc.diff.obs<-summary(H$OU1)$aic.c - summary(H$BM)$aic.c

# Calculate the observed

# aicc differences between more complex and less complex models

######## Model fit tables

fit <- t(sapply(H, function(x) {as.data.frame(rbind(unlist(summary(x)[c("dof", "loglik", "deviance", "aic", "aic.c", "sic")])))}))

print(dat)

print(fit, digits=3)

#############

### Now we generate simulated datasets under BM and habitat models

#############

bm.sim<-simulate(H$BM,nsim=2000) # 200 datasets based on fitted BM model

habitat.sim<-simulate(H$OU1,nsim=2000) # should do 2000 for publication

#############

## Then we fit each simulation under each of the alternative models to

## to generate aicc, aic, sic differences

## So we have four sets of fits:

## A. BM fits when the data is generated under BM

## B. OUhabitat fits when data is generated under BM

## C. BM fits when data generated under OUhabitat

## D. OUhabitat fits when data generated under OUhabitat

##

## Use A B C D to generate these differences:

## aicc differences OU-BM generated under BM = B - A

## aicc differences OU-BM generated under OU = D - C

#############

#############

## BM - data generated on a BM model, specify # of simulations

## fit each simluation using each of the alternative models.

## You can also test different information criteria

#############

## BM sims fit to BM model

bmfits.bm.sim<-lapply(bm.sim, function(x) update(H$BM, data=x))# applies the model of interest to the simulated data

## get information criteria for each sim

bmaicc.bm<-sapply(bmfits.bm.sim, function(x) summary(x)$aic.c)# get aiccs

## BM sims fit to habitat model

habfits.bm.sim<-lapply(bm.sim, function(x) update(H$OU1, data=x))

haicc.bm<-sapply(habfits.bm.sim, function(x) summary(x)$aic.c)

aicc.diffs.bm<-haicc.bm-bmaicc.bm # aicc differences habfit-BMfit when data

# is generated under BM model

#############

## Habitat fits

## For data generated under a habitat model, fit simluation using each of

## the alternative models.

#############

## Habitat sims fit to BM model

bmfits.hab.sim<-lapply(habitat.sim, function(x) update(H$BM, data=x))

bmaicc.hab<-sapply(bmfits.hab.sim, function(x) summary(x)$aic.c)

## Habitat sims fit to habitat model

habfits.hab.sim<-lapply(habitat.sim, function(x) update(H$OU1, data=x))

haicc.hab<-sapply(habfits.hab.sim, function(x) summary(x)$aic.c)

aicc.diffs.hab<-haicc.hab-bmaicc.hab ## aicc difference between fits with hab model minus bm model when data is generated under hab model

maketransparent <- function( color, fraction) { # function to make colors transparent

col <- col2rgb(color)/255

col <- rgb(col[1], col[2], col[3], alpha = fraction)

return(col)

}

BMcol <- maketransparent("black", .5)

HABcol <- maketransparent("black", .2)

pdf(file="Global optimum.pdf")

par(oma=c(0,0,1,0))

#### AIC.c diffs ####

bmdens<-density(aicc.diffs.bm)

habdens<-density(aicc.diffs.hab)

obsdiff<- aicc.diff.obs

plot(bmdens, lwd=3, xlab="AIC.c difference", main = "")

lines(habdens, col=HABcol, lwd=3)

polygon(bmdens, col=BMcol)

polygon(habdens, col=HABcol)

abline(v= obsdiff, lty="dashed")

legend("topleft" , legend=c("BM", "OU1"), xjust=0, col=c(BMcol, HABcol), lty=1, lwd=4, title="Data-generating model", cex=.75, bty="n")

legend("topright", legend="A", bty="n")

title("Global Optimum vs. Brownian Motion CMA", outer=TRUE, cex=1.5)

dev.off()

#######################################################################

## REPEAT:

## Simulate data with large size difference between habitats

## (generated under a HABITAT model with strong selection)

#######################################################################

dat$size <- c(0,0,0,0,0,0,0,0,0,0,0.303670805,0.229467186,0.343263132,0.365666765,0.518043566,0.329995505,0.409418116,0.384948505,0.458968238,0.413996601

,0.427677058) ## mean in forest =1, mean in grass=10

size <- dat$size

names(size) <- names(dat$regimes) <- dat$nodes ## all objects must be named to keep track of species on rows

############ OUCH analyses

H <- list() ## list to hold model output

H$BM <- brown(size, tree)

H$hab <- hansen(size, tree, dat["regimes"], sqrt.alpha=.5, sigma=1)

aicc.diff.obs<-summary(H$hab)$aic.c - summary(H$BM)$aic.c

aic.diff.obs<-summary(H$hab)$aic - summary(H$BM)$aic

sic.diff.obs<-summary(H$hab)$sic - summary(H$BM)$sic

fit <- t(sapply(H, function(x) {as.data.frame(rbind(unlist(summary(x)[c("dof", "loglik", "deviance", "aic", "aic.c", "sic")])))}))

print(dat)

print(fit, digits=3) ## aicc is pretty wonky, probably tree too small

bm.sim<-simulate(H$BM,nsim=2000)

habitat.sim<-simulate(H$hab,nsim=2000)

bmfits.bm.sim<-lapply(bm.sim, function(x) update(H$BM, data=x))

bmaicc.bm<-sapply(bmfits.bm.sim, function(x) summary(x)$aic.c)

bmaic.bm<-sapply(bmfits.bm.sim, function(x) summary(x)$aic)

bmsic.bm<-sapply(bmfits.bm.sim, function(x) summary(x)$sic)

habfits.bm.sim<-lapply(bm.sim, function(x) update(H$hab, data=x))

haicc.bm<-sapply(habfits.bm.sim, function(x) summary(x)$aic.c)

haic.bm<-sapply(habfits.bm.sim, function(x) summary(x)$aic)

hsic.bm<-sapply(habfits.bm.sim, function(x) summary(x)$sic)

bmfits.hab.sim<-lapply(habitat.sim, function(x) update(H$BM, data=x))

bmaicc.hab<-sapply(bmfits.hab.sim, function(x) summary(x)$aic.c)

bmaic.hab<-sapply(bmfits.hab.sim, function(x) summary(x)$aic)

bmsic.hab<-sapply(bmfits.hab.sim, function(x) summary(x)$sic)

habfits.hab.sim<-lapply(habitat.sim, function(x) update(H$hab, data=x))

haicc.hab<-sapply(habfits.hab.sim, function(x) summary(x)$aic.c)

haic.hab<-sapply(habfits.hab.sim, function(x) summary(x)$aic)

hsic.hab<-sapply(habfits.hab.sim, function(x) summary(x)$sic)

aicc.diffs.bm<-haicc.bm-bmaicc.bm

aicc.diffs.hab<-haicc.hab-bmaicc.hab

aic.diffs.bm<-haic.bm-bmaic.bm

aic.diffs.hab<-haic.hab-bmaic.hab

sic.diffs.bm<-hsic.bm-bmsic.bm

sic.diffs.hab<-hsic.hab-bmsic.hab

############################################

## You can also calculate model selection frequencies

## simulated under the best-fit model

############################################

model.sel <- c(sum(aicc.diffs.hab<0), sum(aic.diffs.hab<0), sum(sic.diffs.hab<0))/length(aicc.diffs.hab)

model.sel <- data.frame(model.sel)

names(model.sel) <- "OU1 model"

row.names(model.sel) <- c("aicc", "aic", "sic")

print(model.sel)

pdf(file="Global optimum_MEH.pdf")

par(oma=c(0,0,1,0))

#### AIC.c diffs ####

bmdens<-density(aicc.diffs.bm)

habdens<-density(aicc.diffs.hab)

obsdiff<- aicc.diff.obs

plot(bmdens, lwd=3, xlab="AIC.c difference", main = "")

lines(habdens, col=HABcol, lwd=3)

polygon(bmdens, col=BMcol)

polygon(habdens, col=HABcol)

abline(v= obsdiff, lty="dashed")

legend(-80,.2 , legend=c("BM", "OU1"), xjust=0, col=c(BMcol, HABcol), lty=1, lwd=4, title="Data-generating model", cex=.75, bty="n")

legend("topright", legend="A", bty="n")

title("BM vs. OU1", outer=TRUE, cex=1.5)

dev.off()

################################################################################################################

##OMA##

################################################################################################################

## simulate some size data

names(OMA)<- names(dat$regimes)<- names(bm)<- dat$nodes ## all objects must be named to keep track of species on rows

plot(tree, regimes=gen_pisc_inv, lwd=5, frame.plot=F) ## plot habitat hypothesis

dat$gen_pisc_inv<- gen_pisc_inv

dat$OMA <- OMA

#######################################################################

############ OUCH analyses

## body size simulated with NO DIFFERENCE between habitats

## Note: This is a TINY tree. If the models donÊ»t converge try simulating

## another size dataset until it converges

#######################################################################

H <- list() ## list to hold model output

H$BM <- brown(OMA, tree)

H$OU1 <- hansen(OMA, tree, regimes =dat["regimes"] , sqrt.alpha=.5, sigma=1)

aicc.diff.obs<-summary(H$OU1)$aic.c - summary(H$BM)$aic.c

# Calculate the observed

# aicc differences between more complex and less complex models

######## Model fit tables

fit <- t(sapply(H, function(x) {as.data.frame(rbind(unlist(summary(x)[c("dof", "loglik", "deviance", "aic", "aic.c", "sic")])))}))

print(dat)

print(fit, digits=3)

#############

### Now we generate simulated datasets under BM and habitat models

#############

bm.sim<-simulate(H$BM,nsim=2000) # 200 datasets based on fitted BM model

habitat.sim<-simulate(H$OU1,nsim=2000) # should do 2000 for publication

#############

## Then we fit each simulation under each of the alternative models to

## to generate aicc, aic, sic differences

## So we have four sets of fits:

## A. BM fits when the data is generated under BM

## B. OUhabitat fits when data is generated under BM

## C. BM fits when data generated under OUhabitat

## D. OUhabitat fits when data generated under OUhabitat

##

## Use A B C D to generate these differences:

## aicc differences OU-BM generated under BM = B - A

## aicc differences OU-BM generated under OU = D - C

#############

#############

## BM - data generated on a BM model, specify # of simulations

## fit each simluation using each of the alternative models.

## You can also test different information criteria

#############

## BM sims fit to BM model

bmfits.bm.sim<-lapply(bm.sim, function(x) update(H$BM, data=x))# applies the model of interest to the simulated data

## get information criteria for each sim

bmaicc.bm<-sapply(bmfits.bm.sim, function(x) summary(x)$aic.c)# get aiccs

## BM sims fit to habitat model

habfits.bm.sim<-lapply(bm.sim, function(x) update(H$OU1, data=x))

haicc.bm<-sapply(habfits.bm.sim, function(x) summary(x)$aic.c)

aicc.diffs.bm<-haicc.bm-bmaicc.bm # aicc differences habfit-BMfit when data

# is generated under BM model

#############

## Habitat fits

## For data generated under a habitat model, fit simluation using each of

## the alternative models.

#############

## Habitat sims fit to BM model

bmfits.hab.sim<-lapply(habitat.sim, function(x) update(H$BM, data=x))

bmaicc.hab<-sapply(bmfits.hab.sim, function(x) summary(x)$aic.c)

## Habitat sims fit to habitat model

habfits.hab.sim<-lapply(habitat.sim, function(x) update(H$OU1, data=x))

haicc.hab<-sapply(habfits.hab.sim, function(x) summary(x)$aic.c)

aicc.diffs.hab<-haicc.hab-bmaicc.hab ## aicc difference between fits with hab model minus bm model when data is generated under hab model

maketransparent <- function( color, fraction) { # function to make colors transparent

col <- col2rgb(color)/255

col <- rgb(col[1], col[2], col[3], alpha = fraction)

return(col)

}

BMcol <- maketransparent("black", .5)

HABcol <- maketransparent("black", .2)

pdf(file="Global optimum_OMA.pdf")

par(oma=c(0,0,1,0))

#### AIC.c diffs ####

bmdens<-density(aicc.diffs.bm)

habdens<-density(aicc.diffs.hab)

obsdiff<- aicc.diff.obs

plot(bmdens, lwd=3, xlab="AIC.c difference", main = "")

lines(habdens, col=HABcol, lwd=3)

polygon(bmdens, col=BMcol)

polygon(habdens, col=HABcol)

abline(v= obsdiff, lty="dashed")

legend("topleft" , legend=c("BM", "OU1"), xjust=0, col=c(BMcol, HABcol), lty=1, lwd=4, title="Data-generating model", cex=.75, bty="n")

legend("topright", legend="A", bty="n")

title("Global Optimum vs. Brownian Motion OMA", outer=TRUE, cex=1.5)

dev.off()

#######################################################################

## REPEAT:

## Simulate data with large size difference between habitats

## (generated under a HABITAT model with strong selection)

#######################################################################

dat$OMA <- c(0,0,0,0,0,0,0,0,0,0,0.286554626,0.221207486,0.359906943,0.365990789,0.500316456,0.663001654,0.445875214,0.309796841,0.572238894,0.40957894,0.509851065

) ## mean in forest =1, mean in grass=10

OMA <- dat$OMA

names(OMA) <- names(dat$regimes) <- dat$nodes ## all objects must be named to keep track of species on rows

############ OUCH analyses

H <- list() ## list to hold model output

H$BM <- brown(OMA, tree)

H$hab <- hansen(OMA, tree, regimes=dat["regimes"], sqrt.alpha=.5, sigma=1)

aicc.diff.obs<-summary(H$hab)$aic.c - summary(H$BM)$aic.c

aic.diff.obs<-summary(H$hab)$aic - summary(H$BM)$aic

sic.diff.obs<-summary(H$hab)$sic - summary(H$BM)$sic

fit <- t(sapply(H, function(x) {as.data.frame(rbind(unlist(summary(x)[c("dof", "loglik", "deviance", "aic", "aic.c", "sic")])))}))

print(dat)

print(fit, digits=3) ## aicc is pretty wonky, probably tree too small

bm.sim<-simulate(H$BM,nsim=2000)

habitat.sim<-simulate(H$hab,nsim=2000)

bmfits.bm.sim<-lapply(bm.sim, function(x) update(H$BM, data=x))

bmaicc.bm<-sapply(bmfits.bm.sim, function(x) summary(x)$aic.c)

bmaic.bm<-sapply(bmfits.bm.sim, function(x) summary(x)$aic)

bmsic.bm<-sapply(bmfits.bm.sim, function(x) summary(x)$sic)

habfits.bm.sim<-lapply(bm.sim, function(x) update(H$hab, data=x))

haicc.bm<-sapply(habfits.bm.sim, function(x) summary(x)$aic.c)

haic.bm<-sapply(habfits.bm.sim, function(x) summary(x)$aic)

hsic.bm<-sapply(habfits.bm.sim, function(x) summary(x)$sic)

bmfits.hab.sim<-lapply(habitat.sim, function(x) update(H$BM, data=x))

bmaicc.hab<-sapply(bmfits.hab.sim, function(x) summary(x)$aic.c)

bmaic.hab<-sapply(bmfits.hab.sim, function(x) summary(x)$aic)

bmsic.hab<-sapply(bmfits.hab.sim, function(x) summary(x)$sic)

habfits.hab.sim<-lapply(habitat.sim, function(x) update(H$hab, data=x))

haicc.hab<-sapply(habfits.hab.sim, function(x) summary(x)$aic.c)

haic.hab<-sapply(habfits.hab.sim, function(x) summary(x)$aic)

hsic.hab<-sapply(habfits.hab.sim, function(x) summary(x)$sic)

aicc.diffs.bm<-haicc.bm-bmaicc.bm

aicc.diffs.hab<-haicc.hab-bmaicc.hab

aic.diffs.bm<-haic.bm-bmaic.bm

aic.diffs.hab<-haic.hab-bmaic.hab

sic.diffs.bm<-hsic.bm-bmsic.bm

sic.diffs.hab<-hsic.hab-bmsic.hab

############################################

## You can also calculate model selection frequencies

## simulated under the best-fit model

############################################

model.sel <- c(sum(aicc.diffs.hab<0), sum(aic.diffs.hab<0), sum(sic.diffs.hab<0))/length(aicc.diffs.hab)

model.sel <- data.frame(model.sel)

names(model.sel) <- "OU1 model"

row.names(model.sel) <- c("aicc", "aic", "sic")

print(model.sel)

pdf(file="OU1_OMA.pdf")

par(oma=c(0,0,1,0))

#### AIC.c diffs ####

bmdens<-density(aicc.diffs.bm)

habdens<-density(aicc.diffs.hab)

obsdiff<- aicc.diff.obs

plot(bmdens, lwd=3, xlab="AIC.c difference", main = "")

lines(habdens, col=HABcol, lwd=3)

polygon(bmdens, col=BMcol)

polygon(habdens, col=HABcol)

abline(v= obsdiff, lty="dashed")

legend(-80,.2 , legend=c("BM", "OU1"), xjust=0, col=c(BMcol, HABcol), lty=1, lwd=4, title="Data-generating model", cex=.75, bty="n")

legend("topright", legend="A", bty="n")

title("BM vs. ou1", outer=TRUE, cex=1.5)

dev.off()
