# Supplementary figures and images for: Ecomorphology of Neotropical Electric Fishes: An Integrative Approach to Testing the Relationships between Form, Function, and Trophic Ecology
Source: Integr Org Biol. 2019 Jul 2;1(1):obz015. doi: 10.1093/iob/obz015 (PMC7671154; doi:10.1093/iob/obz015)

A

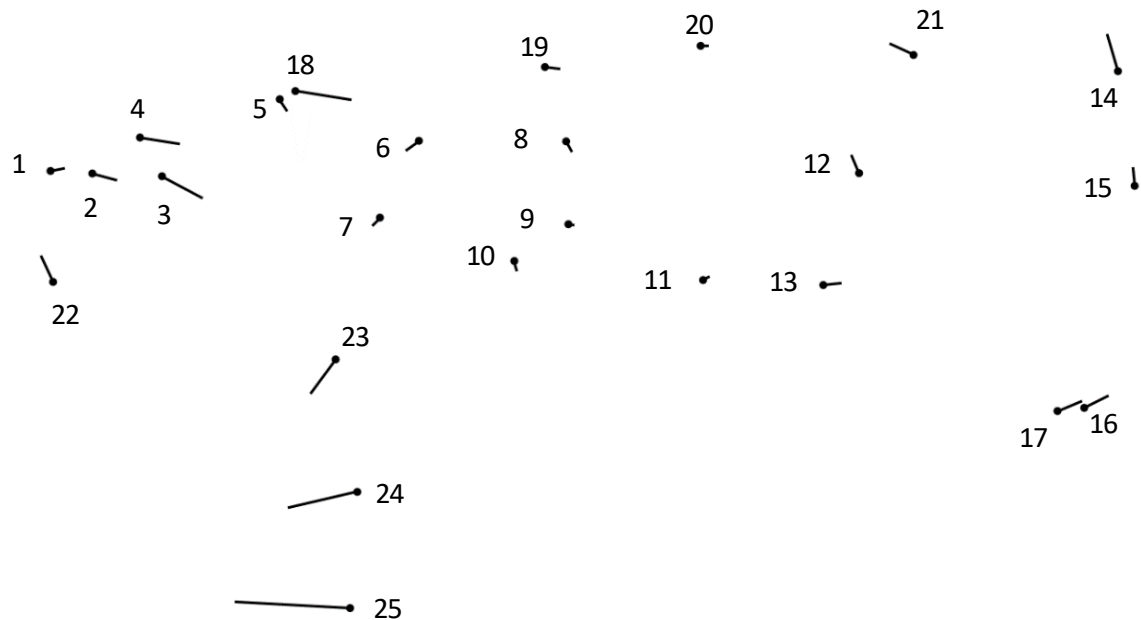

B

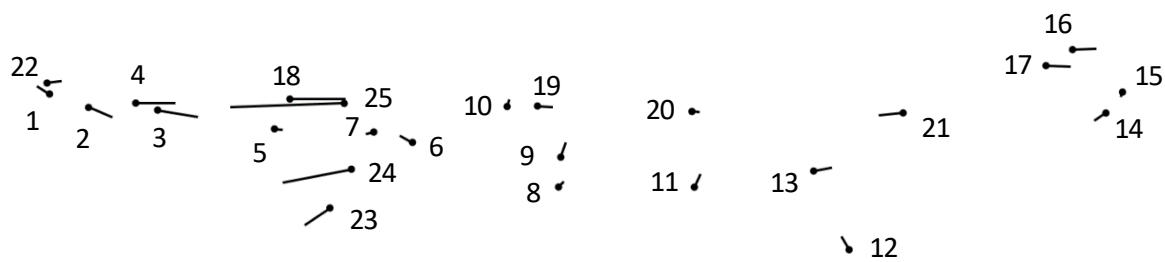

PC1: 40.8%

C

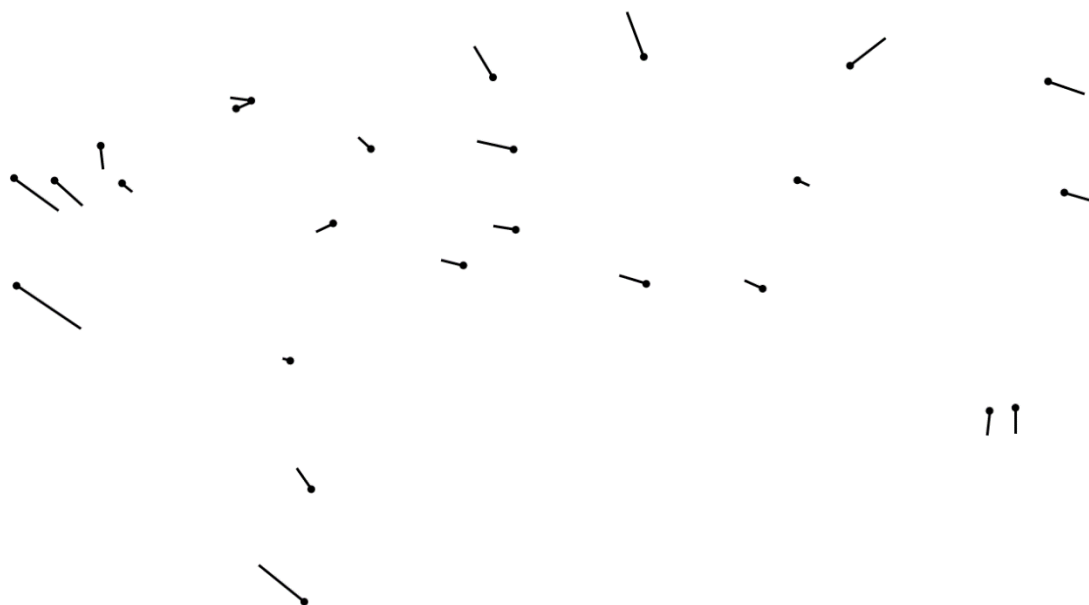

D

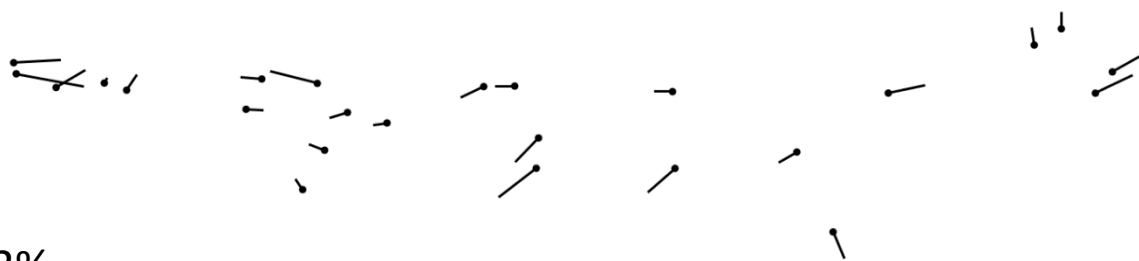

PC2: 27.3%

Supplement: obz015_Supplementary_Data [file obz015_supplementary_data.zip › Supplementary Figure 3.pdf]
